# Role of protected areas in mitigating range loss and local extinctions of terrestrial mammals

**DOI:** 10.1111/cobi.70092

**Published:** 2025-06-16

**Authors:** Andrea Cristiano, Rajeev Pillay, Juan Pablo Ramírez‐Delgado, James E. M. Watson, Oscar Venter, Michela Pacifici

**Affiliations:** ^1^ Department of Biology and Biotechnologies ‘Charles Darwin’ Sapienza University of Rome Rome Italy; ^2^ Department of Geography and Environmental Sciences Northumbria University Newcastle upon Tyne UK; ^3^ Natural Resources and Environmental Studies Institute University of Northern British Columbia Prince George British Columbia Canada; ^4^ Natural Resources and Environmental Studies Graduate Program University of Northern British Columbia Prince George British Columbia Canada; ^5^ School of the Environment The University of Queensland Brisbane Queensland Australia

**Keywords:** anthromes, biodiversity decline, biomes, local extinction, mammal species, matching methods, protected areas, range change, antromas, áreas protegidas, biomas, cambios en la distribución, declinación de la biodiversidad, especies de mamíferos, extinción local, métodos de emparejamiento

## Abstract

Protected areas (PAs) are a major tool in biodiversity conservation, but the extent to which they mitigate species declines is often unclear. We evaluated the effectiveness of PAs in mitigating range contraction and local extinction for 483 terrestrial mammal species. We used expert‐based species range maps from the 1970s and compared them with current distributions to estimate changes in range area and PA coverage over the last 5 decades. We used generalized boosted models and propensity score matching to assess the effectiveness of PAs in mitigating species declines in landscape units sharing environmental features but differing in protection status. On average, mammal species were extirpated across one third of their ranges from 1970 to 2015, and 86% of this range contraction occurred outside PAs. In areas protected continuously since the 1970s, extirpation rates were half that in matched landscape units that were never protected. Areas protected since the 1970s also had lower extirpation rates compared with matched areas that became protected later. Lower species extirpation and range contraction rates were also correlated with greater area encompassed by PAs. Although the proportion of species ranges encompassed by PAs seemingly increased by 12% from 1970 to 2015, this increase may be largely attributed to the loss of species range area outside PAs rather than the strategic expansion of PAs. Our results showed that PAs can mitigate range loss and local extinctions for mammals and highlighted that PAs need to be maintained continuously over long time frames to have positive effects on biodiversity. Our findings suggest that downgrading, downsizing, and degazettement of PAs can lead to biodiversity declines, whereas the strategic achievement of targets, such as protecting 30% of land area by 2030, could facilitate species persistence.

## Abstract (es)

El papel de las áreas protegidas en la mitigación de la pérdida de distribución y las extinciones locales de los mamíferos terrestres

**Resumen:**

Las áreas protegidas (AP) son una herramienta fundamental para la conservación de la biodiversidad, pero a menudo no es claro el punto hasta el que mitigan el declive de las especies. Evaluamos la eficacia de las áreas protegidas para mitigar la contracción del área de distribución y la extinción local de 483 especies de mamíferos terrestres. Utilizamos mapas de distribución de especies de la década de 1970 basados en expertos y los comparamos con las distribuciones actuales para estimar los cambios en el área de distribución y la cobertura de las AP en las últimas cinco décadas. Usamos modelos de impulso generalizado y emparejamiento por puntuación de propensión para evaluar la eficacia de las AP en la mitigación de la disminución de especies en unidades de paisaje que comparten características ambientales, pero difieren en el estado de protección. En promedio, las especies de mamíferos fueron extirpadas en un tercio de sus áreas de distribución entre 1970 y 2015, y el 6% de esta contracción del área de distribución se produjo fuera de las AP. En las zonas con protección continua desde la década de 1970, las tasas de extirpación fueron la mitad que en las unidades de paisaje que nunca estuvieron protegidas. Estas zonas también presentaron tasas menores de extirpación en comparación con las zonas emparejadas que se protegieron más tarde. Las tasas más bajas de extirpación de especies y de contracción del área de distribución tuvieron correlación con una mayor superficie abarcada por las AP. Aunque la proporción de áreas de distribución de especies abarcadas por AP aparentemente aumentó en un 12% desde 1970 hasta 2015, este aumento puede atribuirse en gran medida a la pérdida de área de distribución de especies fuera de las AP en lugar de a la expansión estratégica de las AP. Nuestros resultados muestran que las AP pueden mitigar la pérdida de área de distribución y las extinciones locales de mamíferos y resalta que las AP deben mantenerse de forma continua durante largos periodos de tiempo para tener efectos positivos sobre la biodiversidad. Nuestros hallazgos sugieren que la degradación, la reducción de tamaño y la desregulación de las AP pueden conducir a la disminución de la biodiversidad, mientras que el logro estratégico de objetivos, como la protección del 30% de la superficie terrestre para 2030, podría facilitar la persistencia de las especies.

## INTRODUCTION

Protected areas (PAs) represent a widespread area‐based conservation strategy to avert biodiversity declines (Rodrigues & Cazalis, 2020; Watson et al., 2014). In 2020, PAs covered approximately 22.5 million km^2^ (17% of the world's land surface) (UNEP‐WCMC & IUCN, 2021), with recent targets set to further expand this coverage to 30% of the Earth's land surface by 2030 (Convention on Biological Diversity [CBD], 2022). Given that the creation, maintenance, and expansion of PAs are key components of global environmental policies and conservation efforts (CBD, 2022), it is important to understand the extent to which PAs contribute to actual conservation outcomes such as mitigating species range contractions. Likewise, it is vital to assess the role of PAs in reducing local extinction rates that could otherwise be greater in the absence of protection. Much research on PA effectiveness has focused on the condition of native habitats for biodiversity, extent of anthropogenic pressures, and management capacity rather than the actual status of biodiversity itself (Bruner et al., 2001; Geldmann et al., 2018, 2019; Jones et al., 2018). PAs can provide localized benefits to biodiversity within their boundaries and in immediately adjacent areas by retaining and enhancing species richness, preventing habitat loss, and supporting species persistence and colonization (Brodie et al., 2023; Cazalis et al., 2020; Geldmann et al., 2013; Gillingham et al., 2015; Pillay, Miller, et al., 2022; Thomas et al., 2012). Yet, it is unclear whether these positive effects persist at the larger scales required to support species persistence and range stability in the longer term. Consequently, it remains ambiguous whether PAs are effective at preventing or at least mitigating species range contractions and local extinctions compared with unprotected landscapes with similar environmental conditions (Pacifici, Di Marco, et al., 2020; Williams et al., 2022).

We carried out the first quantitative assessment of the effectiveness of PAs at mitigating range contractions and local extinctions for 483 species of terrestrial mammals for which range limits have been documented from the 1970s through the 1980s. Mammals represent a taxonomic group with reliable ecological and distributional data and have one of the highest percentages of known species (90%) assessed under the International Union for Conservation of Nature (IUCN) Red List criteria (IUCN, 2024). They often serve as indicator species of wider ecosystem functions and status (Brodie et al., 2021; Lacher et al., 2019) and provide relevant examples of the biodiversity crisis, with many considered flagship species of successful conservation programs (Bolam et al., 2021). Our analyses built on prior work that examined how the persistence of many species of mammals is increasingly dependent on the coverage of PAs in their geographic ranges (Pacifici, Rondinini et al., 2020. We expanded on original datasets (Pacifici et al., 2019, 2023) to compare past (1970s) and recent (2015) distributions for 483 species of mammals (Cristiano et al., 2025) and calculated the extent of range change undergone by these species inside and outside PAs over time (UNEP‐WCMC & IUCN, 2020).

We used this dataset to assess the effectiveness of PAs in limiting range contractions and local extinctions of the focal species of mammals, expecting range loss to be less severe for species that are protected over greater proportions of their ranges. We also expected landscape units protected for longer time frames to experience lower extinction rates compared with unprotected units. To assess the effectiveness of PAs in mitigating mammal declines, we used statistical matching methods to isolate the effects of protection against unprotected counterfactuals. We expected protected landscape units would have lower species extinction rates compared with matched units with similar natural and anthropic conditions that were unprotected. We also expected that range loss would be less pronounced for species that experienced an increase in proportional coverage of PAs over their ranges.

By integrating historical data on species ranges, environmental covariates, and different categories of protection status, we sought to provide a novel assessment of range contraction and local extinction for terrestrial mammals inside and outside PAs and to delineate the role of PAs in mitigating biodiversity loss over relatively long time frames.

## METHODS

All spatial analyses described below were performed in World Mollweide (EPSG:54009) coordinate reference system with QGIS 3.22 (QGIS.org, 2021) at a spatial resolution of 0.0833°, corresponding to 5 arc minutes and approximately 10 km at the Equator. Pixel area in all raster analyses was approximately 100 km^2^. Data collected in polygon vector format were converted to raster grids with the default settings of the rasterize (vector to raster) function implemented in QGIS and based on GDAL libraries (details Appendix S1). Data collected in raster grid format at the original resolution were downscaled to a resolution of 5 arc minutes with the Warp (Reproject) function implemented in QGIS with bilinear resampling. All statistical analyses were conducted in R 4.3.1 (R Core Team, 2023). The geospatial data we used are freely available in Figshare (https://figshare.com/s/f0907c5ceb828e2bb4f2) (literature past range maps), Zenodo (https://doi.org/10.5281/zenodo.8362601) (literature past range maps), and Zenodo (https://doi.org/10.5281/zenodo.14901221) (original contribution past range maps, R code, data tables, and Appendices S1 & S2).

### Species range maps

We conducted range change analyses by comparing the extent of past and recent mammal species distributions. Recent range maps were downloaded from the latest species assessments of the IUCN Red List of Threatened Species (IUCN, 2024). Past distribution ranges were available for 263 terrestrial nonvolant mammal species from the 1970s to the 1980s (Pacifici et al., 2019, 2023), which we complemented with maps for 32 species of bats from the literature (Pacifici et al., 2020). Following the protocols of Pacifici et al. (2019), we included range maps from the 1980s to the early 1990s for 246 African mammals from the African Mammal Databank (Boitani et al., 1999), which were reviewed to reflect taxonomic changes that have occurred over the last 2 decades. Our complete dataset comprised past range maps for 483 species (full list of species sampled in Appendix S20 in Cristiano et al. [2025]) compiled (1965–1980s) and validated by species experts (Appendix S1) in compliance with the most up‐to‐date *Mapping Standards and Data Quality for IUCN Red List Spatial Data* (IUCN, 2021).

To ensure that our species sample was representative of mammal taxonomic and geographic diversity, we calculated species representation as the ratio of the sampled species co‐occurring in the recent period in each pixel to the number of extant mammal species known to occur in the same pixels (Appendix S11). We overlapped past and recent distribution maps to extract the extent of range change for each species, identifying areas of range lost, retained, and gained from the 1970s to the 2020s (Cristiano et al., 2025). We then calculated local mammal species richness for both past and present as the sum of mammal species co‐occurring in each 100‐km^2^ pixel (the spatial unit in this study) (Appendix S12). We compared richness between the past and present with estimated local rates of species loss or extinctions. Species loss rates represented local extinctions rates, defined as the ratio of the number of species potentially lost from each spatial unit in 2015 to the number of species that were present in the same units in 1970. In estimating extinction rates as such, we assumed that complete range contraction for a species in a pixel meant the extirpation and local extinction of that species from that pixel.

### Protected areas

Global data on terrestrial PAs were obtained from the World Database on Protected Areas (WDPA; UNEP‐WCMC & IUCN, 2020). The WDPA contains data for approximately 295,000 PAs and other effective area‐based conservation measures in 248 countries and territories. It is periodically updated to include newly established sites under IUCN protection categories, and additions are internally validated by data curators in collaboration with original data providers (UNEP‐WCMC, 2019). PAs are classified by their management objectives and include strict Nature Reserves (Ia), Wilderness Areas (Ib), National Parks (II), Natural Monuments or Features (III), habitat and species management areas (IV), protected landscapes or seascapes (V), and PAs with sustainable use of natural resources (VI) (Dudley et al., 2008). Following WDPA guidelines, we considered only PAs that we confirmed as currently existing and that had been officially assigned to an IUCN category (UNEP‐WCMC, 2019). PAs were represented either by polygons showing the extent of their established boundaries or by centroids associated with the known extent of PAs in square kilometers. For PAs with no polygon available to define their boundaries, we processed centroids by demarcating a radial area proportional to the extent of the relative PA (Venter et al., 2014) (radius equal = square root of the PA's reported extent in square kilometers divided by π). PAs without polygon boundaries, centroids, or any other kind of spatially explicit information were excluded from the analyses.

We used the years 1970 and 2015 as reference years for past and recent distribution range maps of species and to quantify change in the extent of PAs. We selected 1970 as the reference year to represent past mammal distributions because most of our past range maps are from the 1965–1980 period (Pacifici et al., 2019; Pacifici, Rondinini, et al., 2020). We selected 2015 as the baseline to represent the most recently known mammal distribution ranges because most of the species in our dataset were effectively assessed from 2013 to 2015 (IUCN, 2024). For these periods, we identified 3 different protection time frames (Appendix S13): PAs in continuous existence since the 1970s (i.e., PAs established before 1970 and still in existence); PAs existing as of 2015 but that were established after 1970; and other remaining areas that had never been protected. Additionally, we delineated an area with a maximum distance of 10 km (equivalent to one pixel) from the boundaries of all PAs and used this area to assess potential spillover effects between PAs and the unprotected areas outside their boundaries.

### Variables for statistical matching

To limit the potential for landscape‐level environmental and anthropogenic variation confounding the effects of PAs on mammal species richness and range dynamics, we identified pixel‐level landscape units at a 100‐km^2^ resolution (Pacifici et al., 2020) with matching landscape features but differing in protection status over time. We did so by merging the world's terrestrial biomes (aggregated ecoregions representing natural landscapes [Dinerstetin et al., 2017]) and anthromes (aggregated anthropogenic land‐use classes [Ellis & Ramankutty, 2008; Goldewijk et al., 2017]) with gridded data on elevation, slope, human population density, and travel time to the nearest city. These variables were used to match landscape units with similar landscape features but differing in protection status (UNEP‐WCMC & IUCN, 2020).

We obtained elevation and slope data from EarthEnv, a global topography raster dataset based on digital elevation models from SRTM4.1 and GMTED2010 (Amatulli et al., 2018), aggregated at a native 10‐km spatial resolution (100‐km^2^ pixels). Human population density was obtained from the first version of the Global Population Density Grid Time Series Estimates, a raster dataset estimating population levels based on censuses and housing data from 1970 to 2000 (CIESIN, Columbia University, 2017). We extracted population density estimates for the year 1970 and used bilinear resampling in QGIS to downscale the raster native resolution of 30 arc seconds to 5 arc minutes, harmonizing it with the format of the other covariates.

Travel time to the nearest city was extracted from a global raster map of accessibility (Nelson, 2008). We calculated travel time in minutes to the nearest settlement of at least 50,000 inhabitants in 2000 at a native resolution of 30 arc seconds that we resampled to 5 arc minutes. Data on travel time to the nearest city prior to 2000 are currently not available.

We obtained data on the world's biomes from the Ecoregions2017 database (Dinerstein et al., 2017) as 14 aggregate classes of ecoregions to identify areas sharing similar climatic conditions and assemblages of native flora and fauna. We assumed the distribution and extent of biomes remained consistent over the study period and consequently used the same biome extents for 1970 and 2015. Land‐use categories were obtained from the HYDE 3.2 History Database of the Global Environment (Goldewijk et al., 2017). HYDE is a database of historical human population estimates and land‐use categories from 10000 BCE to 2017 CE. Land‐use categories are defined as anthromes, which represent human land use in 20 classes comprising urban areas, villages, croplands, rangelands, and seminatural and wilderness areas (Ellis & Ramankutty, 2008). We extracted anthrome classes for both 1970 and 2015 corresponding to the past and recent species range maps. We then intersected biomes and anthromes shapefiles to identify 265 bioanthromes, defined as unique combinations of landscape units sharing similar natural and anthropic attributes, and thereafter integrated them into the set of covariates detailed above.

### Statistical matching for spatial units of analyses

We used the integrated dataset of bioanthromes and associated covariates to identify landscape units sharing similar environmental features but differing in protection status (Sze et al., 2022). To ensure that our results were not sensitive to the selection of different methods used to match and categorize protected and unprotected units, we adopted 2 complementary methodologies and compared the consistency of their results: generalized boosted models (GBMs) and propensity score matching (PSM). We used GBMs to compare the effect of multiple levels of protection and PSM for pairwise comparisons of the effects of specific protection levels.

A GBM is a powerful application of gradient boosting, a machine learning technique that builds additive regression models by sequentially fitting multiple decision trees to improve the accuracy of a model (Friedmann, 2002; McCaffrey et al., 2013; Ridgeway, 2007). With GBMs, the effect of a variable is estimated by keeping a set of covariates at all possible fixed values, until the goodness of fit stabilizes, after which it is possible to identify the effects of multilevel treatments between matched sampling units. In our case, the different levels of protection categories comprised areas that have been protected continuously since the 1970s, areas that became protected after the 1970s and before 2015, buffer zones around PAs (regardless of their time of establishment), and unprotected areas. We used the gbm R package (Ridgeway, 2007) to run boosted models predicting the percentage of species lost in each pixel as a function of protection status and the set of covariates. We calibrated 3 models to run with 10,000, 20,000, and 30,000 trees, respectively. The minimum depth of each tree was equal to 2 (meaning up to 2‐way interactions between matching covariates), the incremental learning rate equaled 0.1, and cross‐validation was 5‐fold.

PSM is a statistical technique used to combine a set of confounding variables into a single measure of distance, which is then used to estimate the relative probability of sampling units being classified under various treatments (here, different types of protection status) (McCaffrey et al., 2013; Schleicher et al., 2020; Stuart et al., 2011). Like GBMs, PSM estimates the effect of a treatment between matched sampling units by accounting for and maintaining other confounding variables at a constant value. Unlike GBMs, however, PSM works only for binary treatments. Therefore, we used PSM for pairwise comparisons among 4 different types of protection categories (as specified below) based on matched landscape units.

We performed matching with the MatchIt R package (Stuart et al., 2011) and used the default behavior of the matchit function in which nearest neighbor matching is applied without replacement and without enforcing a caliper size to avoid dropping any observation from the analyses. We used quartile cut points to categorize the continuous variables (elevation, slope, population density, and travel time), which reduces variability in the covariates and helps improve matching balance.

To evaluate the performance of the PSM methods, we assessed covariate balance with standardized mean differences (SMDs) between treatments, which is a widely used metric to evaluate the performance of PSM (Ho et al., 2007; Imai & Ratkovic, 2014; Schleicher et al., 2020; Stuart, 2010). SMD compares the difference in means of a covariate between a control group and a treatment group, scaled by its standard deviation. An SMD value <0.1 (and closer to 0) indicates the covariate is well balanced between the control and treatment groups (i.e., the matching procedure has successfully reduced bias for that covariate). This PSM workflow was adopted to estimate differences in trends of species loss between pairwise comparisons of a treatment and control group in 4 different instances: protected versus unprotected units (*n* = 338,814 matched spatial units) to assess overall PA effect (a); units protected continuously since the 1970s versus units that became protected anytime from 1970 to 2015 (*n* = 16,342 matched spatial units) to assess the time since PA establishment effect (b); protected units versus units in the area around PAs (*n* = 303,011 matched spatial units) to assess spillover effects around PAs (c); and unprotected units versus units in the area around PAs (*n* = 1,381,784 matched spatial units) to assess spillover effects around unprotected sites (d).

For each of these 4 pairwise comparisons, we always assigned the group with the lower level of protection as the control group (in [a] comparison unprotected units; in [b] units protected from 1970 to 2015; in [c] units in the buffers around PAs; in [d] unprotected units). The group with the higher level of protection was assigned to the treatment group (in [a], comparison protected units; in [b], units protected continuously since the 1970s; in [c], protected units; in [d], units in the buffers around PAs).

### Generalized linear models

The statistical matching methods described above were only applicable for pixel‐level units of analyses. In addition to these matching analyses, we also used the focal mammal species ranges as units of analyses to examine associations between PA extent within species ranges and the rates of range contraction. We estimated the proportion of range lost for each species as a function of the extent of range encompassed by PAs, expecting greater PA coverage within the range of a species to be associated with lower extent of range loss or contraction. Given these analyses were at the species level (i.e., over species ranges) rather than at the landscape level (i.e., use of matched pixels), we used a series of generalized linear models (GLMs) to test whether greater PA coverage within species ranges was associated with reduced rates of range contraction. For each mammal species, we estimated the percentage of range lost from the 1970s to 2015 with a linear regression:

(1) $$\mathrm{range}\;\mathrm{lost}\;\left(\%\right)∼\ln\left(\mathrm{proportional}\;\mathrm{change}\;\mathrm{in}\;\mathrm{protected}\;\mathrm{range}\right),$$

where the proportion of range lost by each species was estimated as a function of the proportional change in the extent of PAs within species ranges. Due to the skewed distribution and large variation in proportional change in PA coverage data, we transformed this variable to its natural logarithm to better approximate a normal distribution. In doing so, we added a small constant (0.01 equaling a 1% increase in protection extent) to include all species (*n* = 46) that did not experience any change in the proportional amount of range protected (for which the natural logarithm of zero would otherwise have been undefined).

Given that proportional change in protected range (in Equation 1) may not reflect original protected extents within species ranges, we also estimated the percentage of range lost by mammal species from the 1970s to 2015 with 2 linear regressions formulated as

(2) $$\mathrm{range}\;\mathrm{lost}\;\left(\%\right)∼\;\ln\left(\mathrm{extent}\;\mathrm{of}\;\mathrm{range}\;\mathrm{protected}\;\mathrm{since}\;1970\right)$$

and

(3) $$\begin{array}{ccc} & & \mathrm{range}\;\mathrm{lost}\;\left(\%\right)∼\ln\left(\mathrm{extent}\;\mathrm{of}\;\mathrm{range}\;\mathrm{protected}\;\mathrm{in}\;2015\;\mathrm{but}\right.\\ & & \left.\;\mathrm{not}\;\mathrm{since}\;1970\right), \end{array}$$

where the proportion of range lost by each species was estimated as a function of the extent of range (in square kilometers) protected continuously since 1970 (Equation 2) and the extent of range protected in 2015 but not in 1970 (Equation 3). As before, we accounted for the skewed distribution and large variation in the extent of the protected ranges by transforming these variables to their natural logarithm. Here, we added a small constant of 1 (equivalent to a 1‐km^2^ increase in protection extent) to ensure species with no portion of their ranges protected either in the 1970s (*n* = 42) or in 2015 (*n* = 14) were included in the analyses.

Given our data represented only 483 species of extant mammals, we made inferences about the effectiveness of PAs in mitigating declines only for a relatively small sample. Extrapolating our results to the entirety of mammal diversity would require the assumption that spatial responses (i.e., range changes) are relatively uniform within the entire class Mammalia. Under this assumption, there would be no observable patterns of range responses across smaller taxonomic units (such as orders or families) or functional groups. Because this assumption is untested, we also checked for possible associations between range changes and ecological traits to ensure that our observed patterns were not driven by any predominant attributes that characterize the species included in our sample. To do so, we ran 2 GLMs to assess whether changes in range area and protected coverage were associated with species’ ecological and life‐history traits (Appendix S3).

Finally, we used bioanthromes as sampling units to test whether an increase in PA coverage in bioanthromes was associated with lower rates of extirpations from the 1970s to 2015. We overlapped bioanthromes with the maps of past, recent, and lost mammal species’ ranges and calculated rates of species loss across those. We then superimposed bioanthromes with all 3 categories of protection time frames and calculated the proportional change in protection from the 1970s to 2015 (i.e., the proportion of each landscape unit that was either protected or unprotected over time). This allowed us to estimate local extinctions with 2 linear regressions formulated as

(4) $$\begin{array}{ccc} & & \mathrm{species}\;\mathrm{locally}\;\mathrm{extinct}\;\left(\%\right)∼\mathrm{change}\;\mathrm{in}\;\mathrm{PA}\;\mathrm{coverage}\\ & & +\mathrm{bioanthrome}\;\mathrm{area}, \end{array}$$

where the percentage of species locally extinct was estimated as a function of proportional change in the coverage of PAs across bioanthromes and bioanthrome area—either in square kilometers or in its natural logarithm.

## RESULTS

### Species richness and sample coverage

The average mammal richness of our sample was 8.9 species in each 100‐km^2^ pixel (range 0–69 species in each pixel) (spatial variation in richness of sampled mammals worldwide in Appendix S12). Our sample represented 8.3% of the taxonomic diversity of extant terrestrial mammal species (Appendix S6) and approximately 28.6% of the taxonomic diversity across the 26 existing mammalian orders. Our sample also represented an average 18.6% of the geographic diversity of terrestrial mammals. This percentage of geographic diversity was calculated across all grid cells included in the study, as the average proportion of species represented in our sample over the entire diversity of terrestrial mammal species (Appendix S11). As such, it was not equivalent to the overall representation of the taxonomic group (8.3%). It was rather the proportional representation of species being sampled in each pixel (i.e., geographic representation). Some biogeographic regions tended to be overrepresented in our dataset (spatial variation in taxonomic representation worldwide in Appendix S11). For instance, the average pixel‐level representation of sampled species was approximately 30% in the Nearctic and 50% in the Afrotropical realms. The Neotropical and Indomalayan realms, which have high mammal diversity (more than 60% of extant mammal species as estimated by Pillay, Venter, et al., 2022), were less represented in terms of average species coverage at the pixel level (approximately 10%).

### Summary of changes in range area, species richness, and protection over time

**FIGURE 1:**
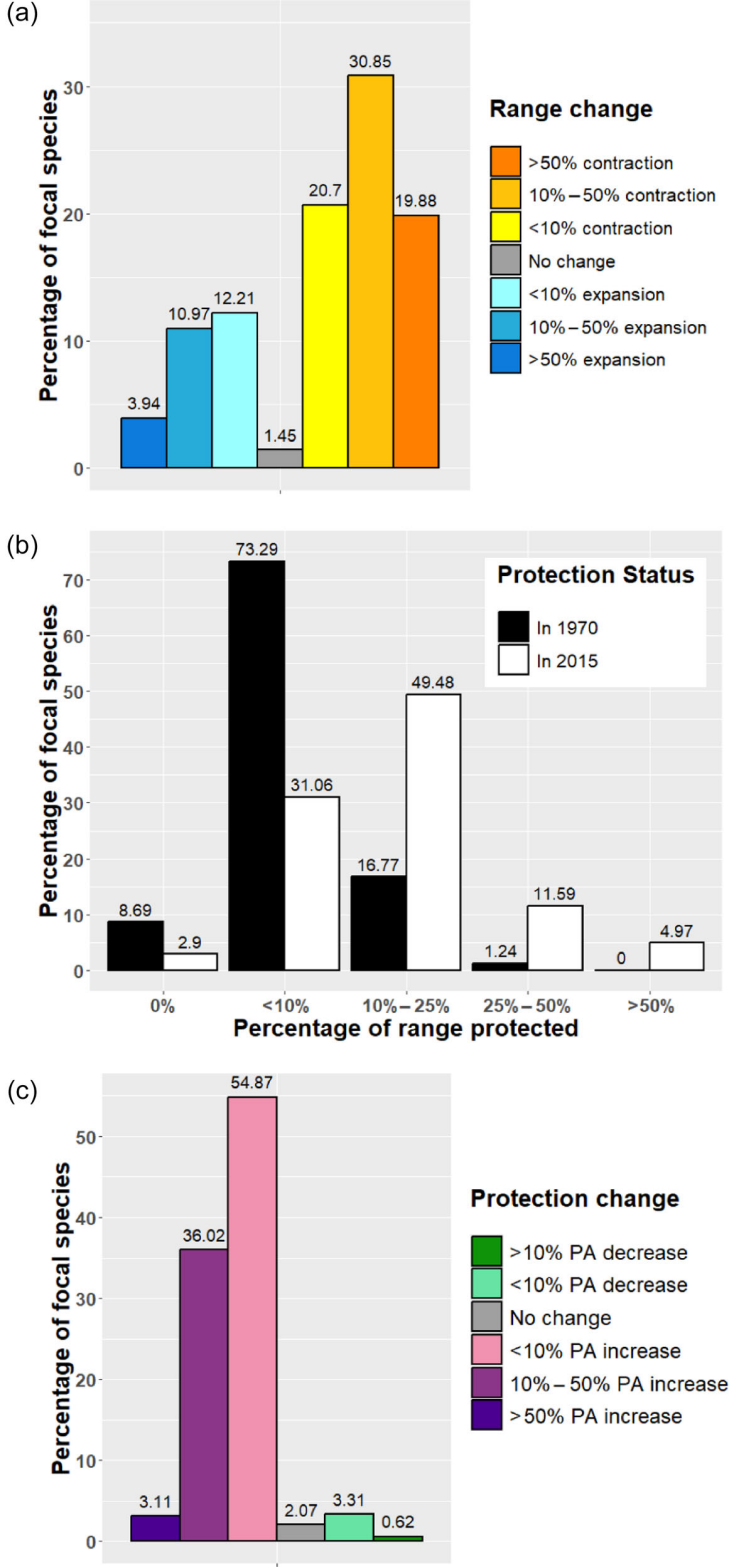
Percentage of species that had (a) range contractions, expansions, or no change in range, (b) change in the number of species as a function of the percentage of range protected over time, and (c) change in the proportional range coverage of protected areas (PA) from the 1970s to 2015.

Our analyses of range changes revealed that the focal species of mammals lost, on average, nearly one third (31.97%) of their original range area from 1970 to 2015 both inside and outside PAs. Only 7 species (1.4% of our sample) did not show any change when comparing their past and recent distribution maps. Of the remaining species, more than two thirds (*n* = 345) experienced net range contraction from 1970 to 2015, with around 20% of focal species losing more than 50% of their original range area. Conversely, less than one third of species (*n* = 131) experienced net range expansion, with the majority of expansions being limited in terms of area because <4% of the focal species gained more than 50% of their original range area (Figure 1a).

In the 1970s, the ranges of 42 species (8.6% of the sample) were completely outside PAs. However, this declined to only 14 species in 2015 (Figure 1b). Similarly, the number of species with <10% of their range protected declined from 354 to 150, whereas the number of species with larger percentages of their range protected increased over time (Figure 1b). Based on estimates of species’ range change and changes in PA extent, the average proportion of species’ ranges encompassed by PAs appeared to increase by approximately 12% (from 5.3% in the 1970s to 17.5% in 2015).

Of the 483 sampled mammal species, 94% experienced some degree of increase in the proportional coverage of PAs over their range. Only 10 species that had completely unprotected ranges in the 1970s remained unprotected in 2015 (Figure 1c). When considering the total range area lost across all species, most of this loss (85.5%) occurred in areas that had never been protected. Conversely, 12.2% of range loss occurred in areas that were not protected in the 1970s but became protected before 2015, and only 4.4% of range loss occurred in areas protected continuously from the 1970s (Appendix S1). The percentages do not add up to 100% because ranges expansions may have occurred in any of the 3 types of protection time frames. Averaged across species, sampled mammals lost approximately one third (32.3%) of their range area that had never been protected and lost approximately one fourth (26.3%) of their range areas that were originally protected since the 1970s (Appendix S1).

**FIGURE 2:**
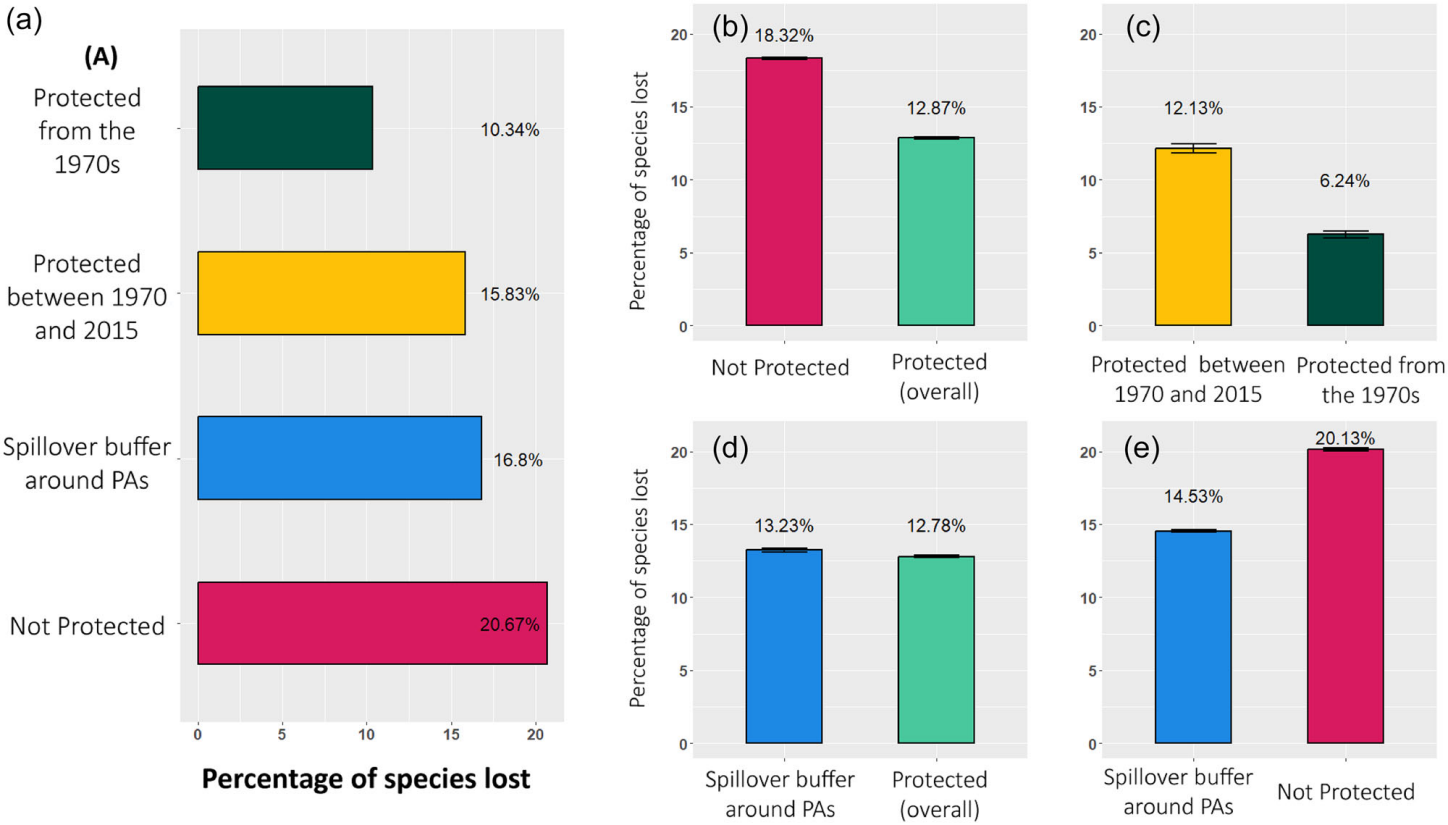
Differences in rates of species loss across matched landscape units with different protection statuses based on (a) generalized boosted models (values, average effect of protection status on sampling units) and (b–e) propensity score matching (values, average marginal estimates of the effect of each binary treatment [protection categories] and 95% confidence intervals).

**FIGURE 3:**
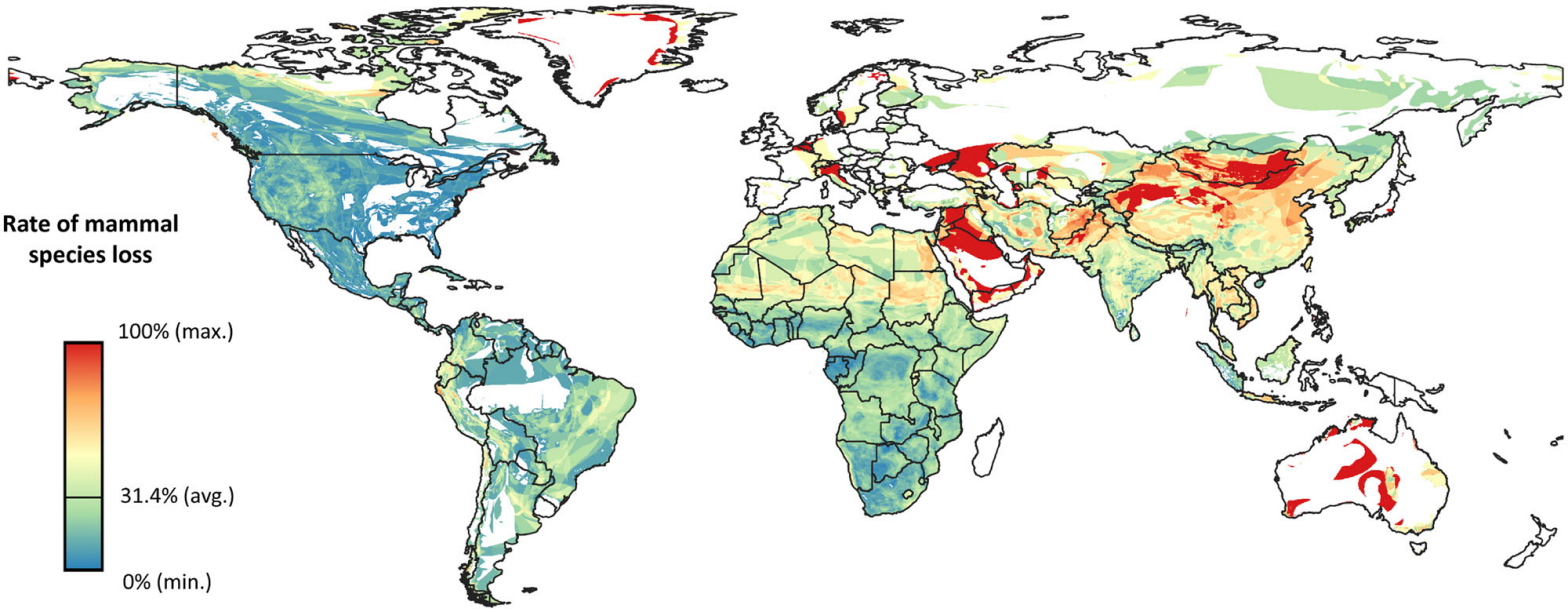
Percentage of species loss from 1970 to 2015 calculated for each pixel as the ratio of sample species extirpated in 2015 against the number of sample species present in 1970 (white, no coverage of species’ range and subsequently no calculated rates of species loss; scale resolution, 0.0833° [equivalent to 5 arc minutes and approximately 10 km at the Equator]; pixel area, approximately 100 km^2^).

Average and maximum species richness of the focal mammals we considered were higher in 1970 than in 2015 (Appendix S12), and differences (95% confidence intervals do not overlap each other) in the percentages of species lost across the different categories of protection differed significantly (Figure 2). Averaged over the world's terrestrial surface, as of 2015 each 100‐km^2^ pixel lost almost one third of the original sampled mammal species richness of the 1970s, with an average local extinction rate of 31.4% albeit with large spatial variation across biogeographical realms (Figure 3).

### Extinction rates and range loss as a function of protection

The outputs of GBMs and PSM were consistent with each other, indicating a positive role of PAs in mitigating local extinction for mammal species. The GBM performance peaked and ceased to improve with 17,537 trees, and the covariate with the highest relative importance to achieve model balance was bioanthromes, showing a large degree of variability in the effect of protection status across different bioanthromes (Appendix S14). On average, species loss was almost half as low in areas protected continuously since the 1970s (10.3%) compared with areas that were never protected (20.7%) (Figure 2a). Areas not protected in 1970s but that became protected as of 2015 were relatively less effective in reducing species loss (15.8%). Their performance was comparable to that of zones around PAs used to account for spillover effects (16.8%). Results of the PSM analyses also supported these findings based on all pairwise comparisons considered. Matching improved the balance of covariates in all cases except for the comparison between protected landscape units and matched buffer zones (Table 1; Appendices S15 & S21). Rates of species loss were lower in PAs than in unprotected areas (Figure 2b) and lower in areas protected from the 1970s than in areas that became protected later (Figure 2c). The matching analyses did not detect a significant difference in rates of species loss between PAs and in the zones around them (Figure 2d), which can be attributed either to potential spillover effects from nearby protected sites or to suboptimal performance of the matching process because this was the only matching comparison for which SMD values of some covariates were not balanced. Moreover, rates of species loss were significantly lower in the buffer around PAs compared with completely unprotected sites (Figure 2e). Although these estimates may underline the presence of spillover effects, we exercised caution in assessing their role and prevalence by considering the suboptimal performance of PSM between PAs and buffer areas around them.

**TABLE 1:** Propensity score matching (PSM) quality showing the balance of continuous covariates after matching based on absolute values of standardized mean difference (SMD) for every iteration of the PSM.

Model parameter	Protected vs. unprotected		Protected from 1970 vs. protected from 1970 to 2015		Spillover area vs. protected		Spillover area vs. unprotected	
	SMD before	SMD after	SMD before	SMD after	SMD before	SMD after	SMD before	SMD after
Distance (propensity score)	0.6142	0.0108	0.6996	0.0631	0.9501	1.2757	0.6661	0.0003
Elevation	0.0562	0.0030	0.0596	0.0266	0.0004	0.0098	0.0595	0.0035
Slope	0.1384	0.0121	0.1492	0.0427	0.0321	0.0484	0.1858	0.0004
Population density	0.1593	0.0041	0.0149	0.0068	0.2787	0.3755	0.0631	0.0014
Travel time to cities	0.2342	0.0061	0.2585	0.0232	0.4686	0.5940	0.5637	0.0045
Bioanthromes (average)	0.0029	<0.0001	0.0048	0.0001	0.0043	0.0056	0.0011	<0.0001

*Note*: Absolute SMD values <0.1 indicate good balance and reliable matching performance. Spill over areas are a maximum of 10 km (equivalent to one pixel) outside the boundaries of protected areas.

**FIGURE 4:**
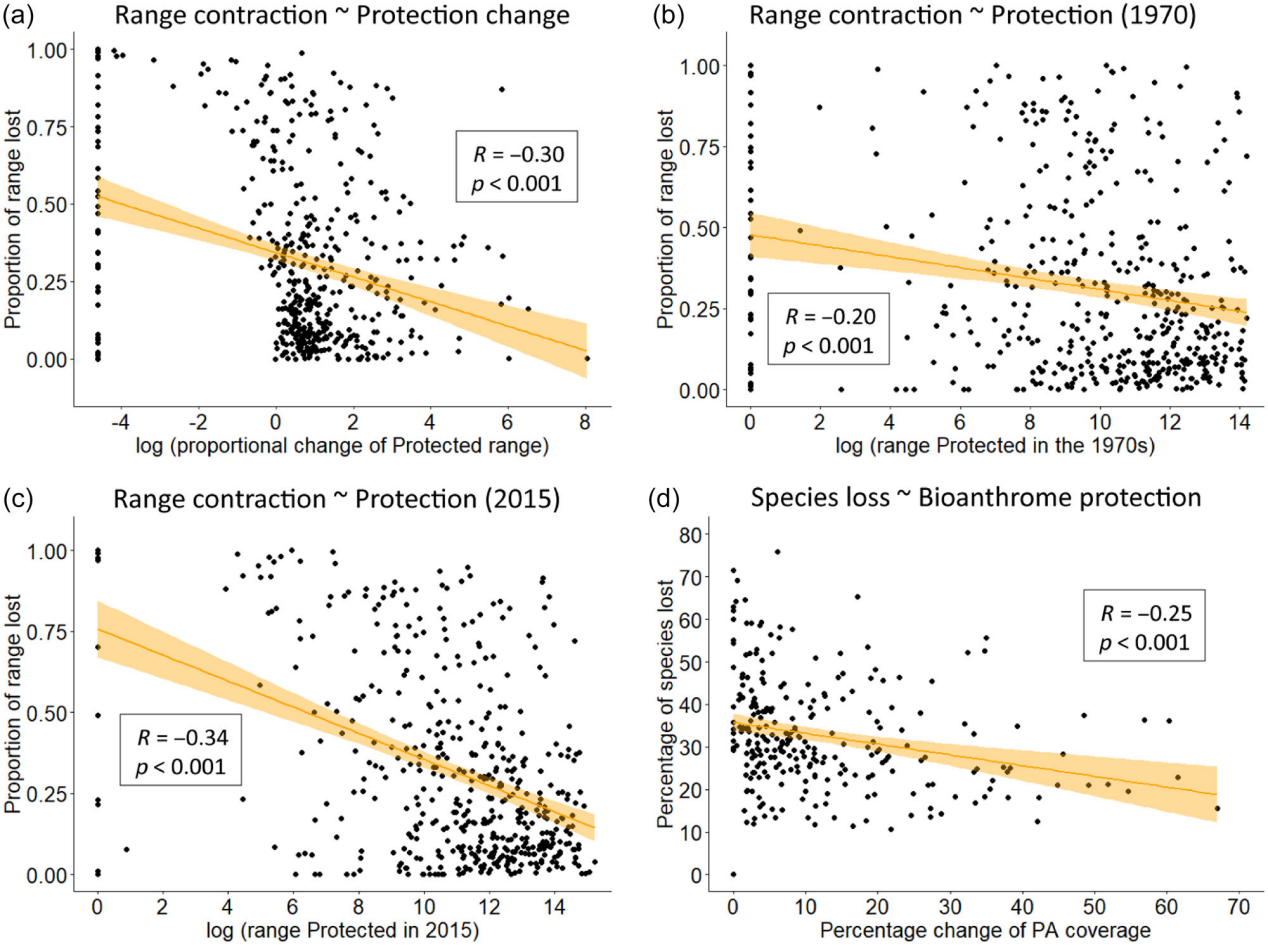
Species‐based associations between species’ range contraction and (a) change in the extent of protection from 1970 to 2015 and extent of range protected from (b) the 1970s and (c) as of 2015 and (d) landscape‐based associations between rates of species loss in each bioanthrome and the proportional change of protected area (PA) coverage (orange, confidence interval of the prediction).

Finally, using species as sampling units in the analysis (Appendix S7), we found that reduced rates of range contraction were significantly associated with increasing proportional coverage of PAs within species’ ranges (Figure 4a) and with larger extents of PA coverage within species’ ranges in the 1970s (Figure 4b) and as of 2015 (Figure 4c). Using bioanthrome categories as units of analysis, we also found that extinction rates decreased significantly as the proportional coverage of PAs increased in each bioanthrome (Figure 4d), and extinction rates were not significantly associated with bioanthrome size (Appendix S8). There was no association between species’ ecological and life‐history traits and species’ range changes or changes in protected coverage.

## DISCUSSION

### Species‐level impacts of PAs

Our results suggest that the long‐term presence of PAs has the potential to mitigate range contraction and local extinctions in terrestrial mammals. Importantly, range contractions were smaller for species that experienced an increase in the proportional coverage of PAs within their ranges. This trend was especially prominent among species that had the most extensive PA coverage, suggesting that increasing the extent of protection over a species’ distribution can contribute to the stability of its geographic range and consequently to the persistence of the species.

For example, with the creation of the Una Biological Reserve in Brazil in the 1980s, the golden‐headed lion tamarin (*Leontopithecus chrysomelas*) experienced an increase of 83% in the proportion of its protected range and lost only 14% of its total geographic range (compared with the average range loss of 32% across all sampled species) despite being an endangered species endemic to the Atlantic Forest, one of the most threatened ecoregions in the world (Oliveira et al., 2021). Similarly, the Tasmanian devil (*Sarcophilus harrisii*) experienced an increase of 41% in the proportion of its protected range, and its overall geographic range remained stable between the 2 periods despite being an endangered species threatened by infectious disease, road mortality, and predation from non‐native species such as dogs and foxes (Hawkins et al., 2008). In such cases, the persistence and expansion of PAs might have facilitated control practices and monitoring programs to mitigate pressures directly threatening these species.

In contrast, most other species in our sample that experienced a decrease in the proportion of their protected range also experienced rates of range loss substantially higher than the sample average. For instance, the giant kangaroo rat (*Dipodomys ingens*), Tapanuli orangutan (*Pongo tapanuliensis*), and New England cottontail (*Sylvilagus transitionalis*) all saw decreases in the proportions of their protected ranges (4–10%, against the average 12% increase in protection of our sample) and experienced range contraction rates of 87–97% (Appendix S1). This would suggest that these species are likely facing intense and widespread pressures that are not limited to unprotected landscapes, and that instead also occur within the boundaries of PAs. Thus, we argue that range loss occurring in PAs may be an indicator of even larger and more generalized species declines.

As ranges shrink due to habitat loss, fragmentation, and degradation, species can become restricted to isolated habitat patches that may or may not be in PAs. In such cases, the processes that lead to range loss (e.g., climate change, competition from invasive species, human disturbance) will likely continue to exert their influence, weakening the overall effectiveness of PAs in providing species with refugia against environmental change.

Our findings might be biased toward taxonomic groups that make up the majority of species represented in our sample. Approximately 75% of our dataset was composed of the orders Carnivora, Cetartiodactyla, Primates, and Rodentia, whereas other orders were underrepresented (e.g., Chiroptera and Eulipotyphla). In the case of rodents, although they comprise 72 of the total 483 sampled species, we could only represent 3% of the taxonomic diversity of the order. Consequently, extrapolating patterns observed in our subset of mammal species to the diversity of all existing mammals still presents significant challenges and may not be recommended, but could only be overcome by integrating more information on past distribution of more species.

Interspecific variability is expected to play a substantial role in driving spatial responses to landscape modifications. However, in our models assessing the associations between range changes and ecological traits, we did not find any significant correlation, suggesting no ecological trait is a clear driver of range change for the sampled species in this study (Beissinger & Riddell, 2021). That said, the lack of signal with species traits in this study warrants further investigation into the mechanisms driving range responses to environmental change. Species that are functionally and phylogenetically distant from each other may be expected to show variable range changes, unless the impact of anthropogenic activities would be so strong as to mask interspecific variability in responses (Di Marco & Santini, 2015). Ideally, such assessments should be based on the highest possible number of species. However, reliable spatial data for the past decades remain sparse, making such conservation assessments challenging.

We compiled past species distribution maps following the rationale and guidelines defined for current IUCN Red List species assessments, which allowed us to compare past and present distributions. Yet, integration of new species occurrence data, following recent advances in methods and techniques to record species occurrence, can substantially affect the outcome of new assessments of species’ ranges change (Elith et al., 2006; Marsh et al., 2023; Tingley & Beissinger, 2009).

We also note that elements of subjectivity, tradition, and culture can play an important role in how ranges are assessed over time. Although the fundamental guidelines and methodologies used in quantitative range assessment may remain broadly consistent, the specific way they are applied on the ground can vary according to the experience and perspective of assessors (Willis et al., 2007). These shift in approaches can often results in improved data quality and resolutions but can also introduce a substantial source of variability in how biodiversity is assessed (Boakes et al., 2010). Such variability can arise over time because of differences in how expertise is gained depending on the social and cultural contexts, and also because of changing paradigms in the scientific community about how certain elements of biodiversity are to be interpreted (Soga & Gaston, 2018). As such, comparing species’ range assessments that are decades apart, even with the same formal methodology, does not necessarily guarantee uniformity of judgments, especially in cases where shifting perspectives and implicit biases result in changing conventions of how biodiversity values are perceived and measured through time. Nevertheless, comparative assessments of species’ range status over time hold considerable value for conservation as they allow inferences on temporal trends based on validated, standardized, spatially explicit data. Future work should explore how patterns of change in species’ ranges change over time. Subsequent assessment of species’ conservation status over time can be influenced not only by emergent methodologies underlying the collection and usage of available data, but also by aspects associated with human behavior, such as changes in conservation priorities or shifting paradigms in human relationships with nature.

### Effectiveness of PAs compared with matched landscape units

After controlling statistically for natural and anthropogenic variation in matched landscape units that differed only in protection status, we found the long‐term presence of PAs can facilitate the persistence of the focal mammal species in this study by limiting extirpation rates at the landscape level. The reduced rates of species loss observed across a gradient of protection categories (from those that have been protected for more than 50 years to those that have never been protected) were consistent between 2 methodologies used to assess treatment effects. Also consistent with our expectations, landscape units that have been protected since the 1970s were associated with extirpation rates that were almost half those of completely unprotected areas. The effect of PAs in reducing species loss was also positive for landscape units that became protected after 1970 and 2015, albeit less pronounced (depending on the methods used) compared with areas protected since the 1970s (Figure 2).

Our results indicated that time since the establishment of PAs is a crucial factor in their effectiveness in conserving biodiversity, with the effects of protection less prominent for more recently established PAs compared with those that have been protecting species and their habitats for longer. Although we found potential evidence for beneficial spillover effects of PAs (with almost no difference in the proportion of species lost in PAs and the buffer zones around their boundaries, and a greater proportion of species lost in unprotected areas compared with buffer zones around PAs), we exercise caution in confirming their role and prevalence because the observed patterns of reduced extirpations in zones around PAs may also be explained by other factors, such as landscape configuration. For example, regardless of the protection status, areas that are close to each other (inside PAs, outside PAs, or at the boundaries between unprotected and protected zones) are likely to share similar landscape configuration and habitat types, and consequently similar species richness and diversity. In such cases, potential positive spillover in zones around PAs might simply be an artifact of the characterization of landscape features and biological communities that occur on those regions, rather than a direct effect of protection status. Although the pairwise comparison between buffer zones and completely unprotected areas revealed the latter to be more exposed to extirpation, future analyses should isolate the presence of spillover effects at the edge between unprotected and PAs, considering other spatially explicit factors, such as the extent of PAs around which spillover buffers are considered, and differences in the composition of biological communities in the regions of study.

Additionally, the pairwise comparison between PAs and adjacent bufferzones remained unbalanced even after matching, indicating residual imbalance from the matching procedure between treatment and control groups, which remained different on the covariates of population density and time travel. Because the performance of boosted models and matching methods remained good across all the other comparisons between protection treatments, including all robustness checks (Appendix S5), we argue that our main results were not driven by contamination from areas outside PAs potentially affected by spillover, rather than inferring the prevalence of such effects.

Together with the positive effects of PA extent within species’ ranges and reduced rates of range contraction, our findings indicated that many of the focal mammal species in our study may have benefitted from the increasing degree of protection over the last 5 decades. Bioanthromes had a strong influence in achieving model balance (Appendix S14), which suggests considerable variation in PA performance depending on natural and anthropogenic landscape characteristics (Appendix S2). For instance, in regions of the world characterized by large‐scale human activities (Watson et al., 2023), the effect of protection may be masked by external factors—such as localized extreme weather events associated with climate change, pollution leakage from urban or agricultural areas, or the presence and expansion of non‐native invasive species (Elsen et al., 2020; McDonald et al., 2009; Moodley et al., 2020; Thomas & Gillingham, 2015). In such cases, the high degree of anthropogenic disturbance can undermine the role of PAs in conserving species where the underlying ecological conditions are already the result of past environmental perturbations (Carroll et al., 2004; Geldmann et al., 2019).

### Implications for conservation planning

Our results highlight the role of PAs in buffering mammals from anthropogenic disturbances, although they are somewhat sensitive to the composition of our focal species. Some areas of the world tend to be better documented in terms of data availability for resident mammal species, a legacy of colonial and post‐colonial mechanisms in conservation practice (Garland, 2008; Grove, 2017; Salomon et al., 2018). Many species of high conservation concern (e.g., Primates) remain relatively less documented despite being severely threatened (IUCN, 2024). Further, species that are relatively uncharismatic, cryptic, or elusive remain poorly studied despite comprising a major fraction of existing mammal diversity (e.g., Chiroptera, Eulipotyphla, Rodentia). As such, any study that tries to document the effectiveness of PAs in conserving mammals must deal with gaps in data that characterize our current comprehension of this heterogeneous group. Additionally, factors such as policy, planning, and management strategies can also affect the performance of PAs at conserving biodiversity (Andrade & Rhodes, 2012; Pressey et al., 2015; Rodrigues & Cazalis, 2020).

PAs alone cannot address the challenges posed by global change to biodiversity, but our findings strongly suggest they are an important conservation tool for mammals. Wherever possible, their performance should be periodically assessed using quantitative metrics, such as rates of range contraction and extirpation, which can provide species‐ and community‐level indicators of the effectiveness of PAs in limiting biodiversity loss. Because PAs are likely to remain the cornerstone of global conservation efforts (CBD, 2022), providing adequate financing and sociopolitical support to the maintenance, connection, and expansion of protected landscapes will be key for the persistence of many mammal species.

## AUTHOR CONTRIBUTIONS

Andrea Cristiano, Oscar Venter, James E. M. Watson, and Michela Pacifici conceived the original idea of the study. Andrea Cristiano and Juan Pablo Ramírez‐Delgado carried out data elaboration. Andrea Cristiano carried out geospatial processing and statistical analyses. Andrea Cristiano, Rajeev Pillay, and Michela Pacifici developed the body of the manuscript. All authors reviewed and corrected the manuscript.

## Supporting information



Figure S1

Figure S2

Figure S3

Figure S4

Figure S5

Figure S6

Figure S7

Figure S8

Figure S9

Supporting Information
