# Supplementary figures and images for: Role of protected areas in mitigating range loss and local extinctions of terrestrial mammals
Source: Conserv Biol. 2025 Jun 16;39(6):e70092. doi: 10.1111/cobi.70092 (PMC12658939; doi:10.1111/cobi.70092)

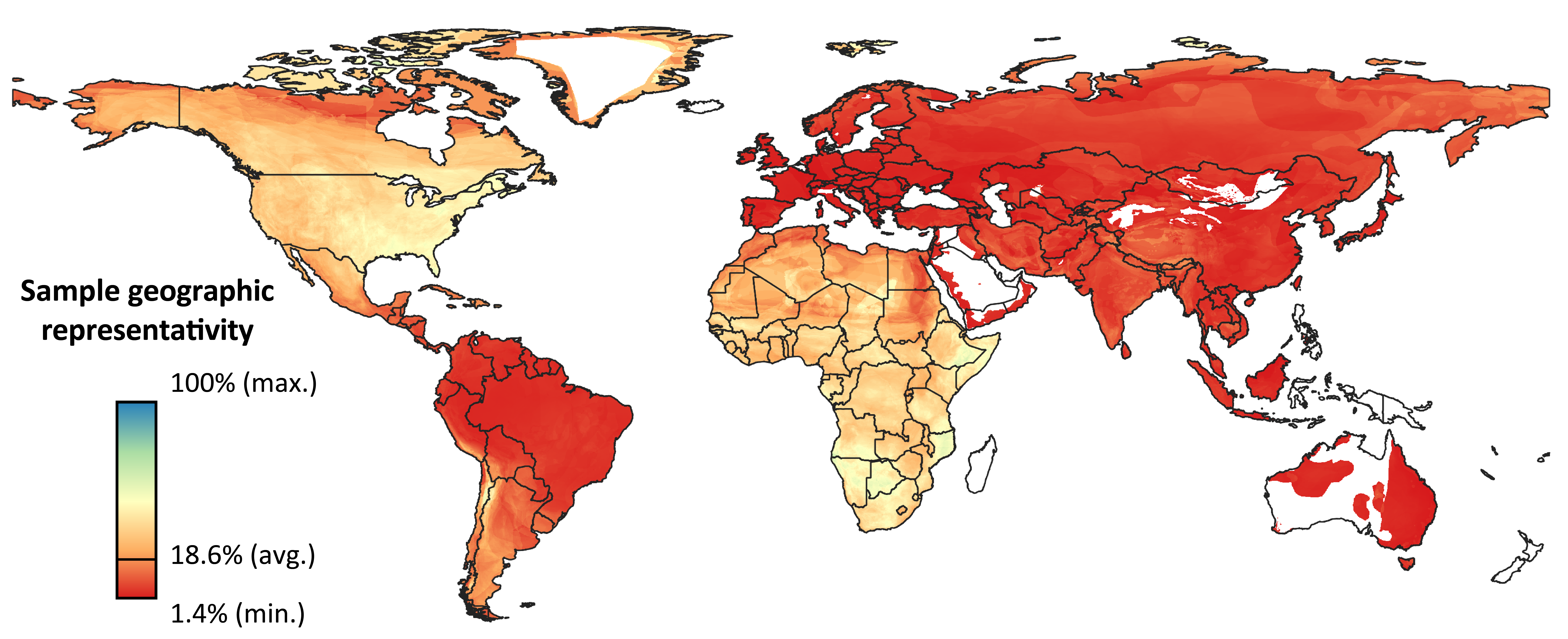

Supplement: Supplementary file 1 — Figure S1 [file COBI-39-e70092-s010.png]

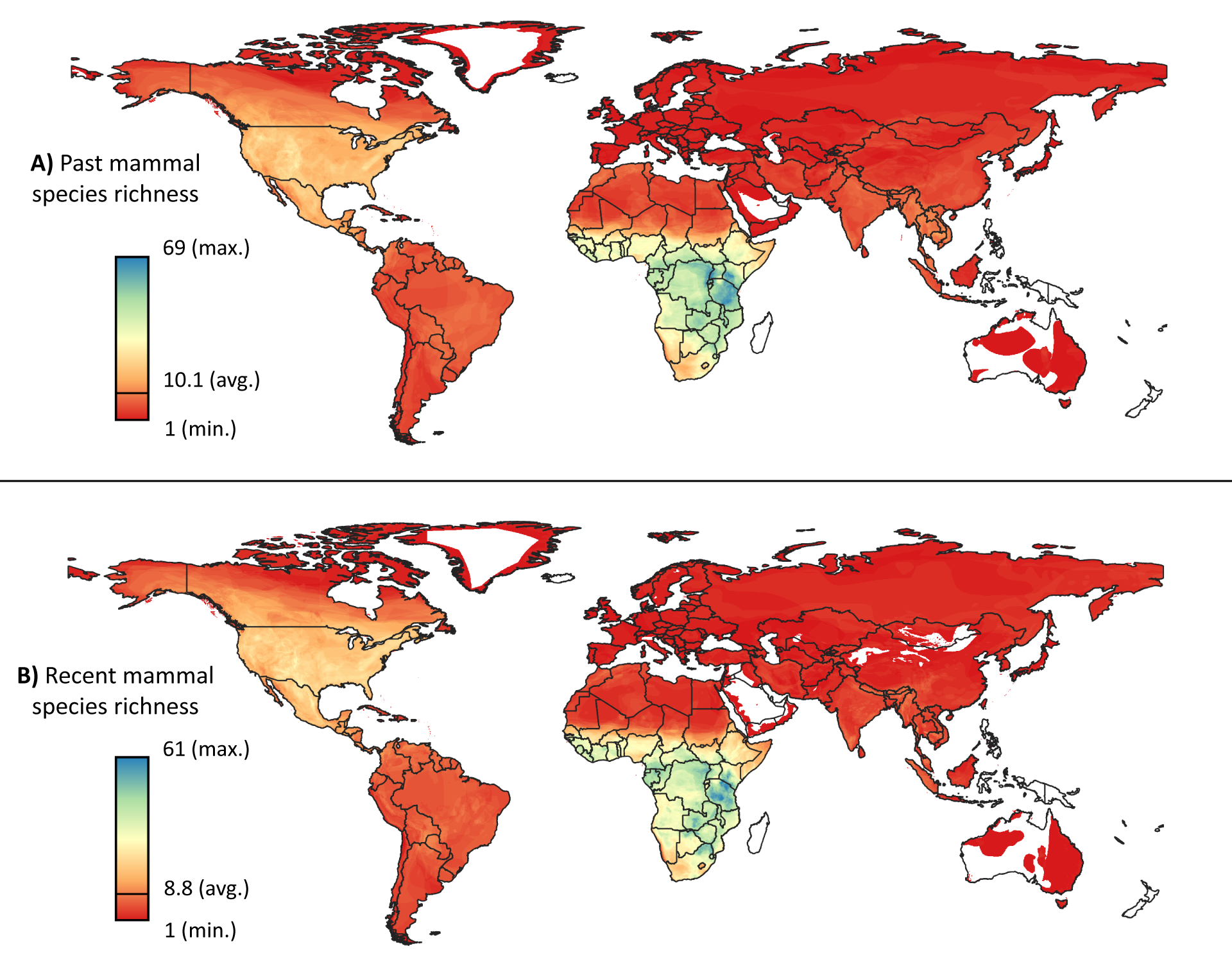

Supplement: Supplementary file 2 — Figure S2 [file COBI-39-e70092-s003.png]

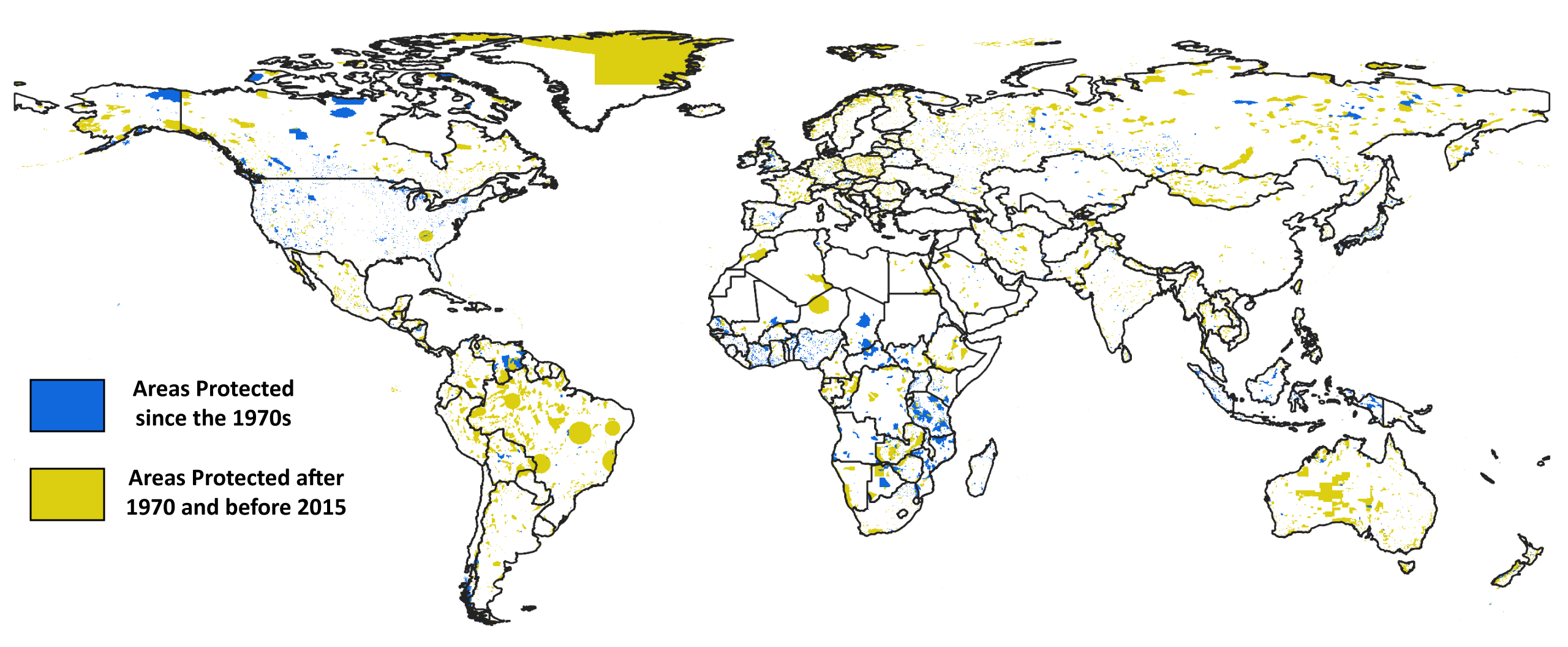

Supplement: Supplementary file 3 — Figure S3 [file COBI-39-e70092-s001.png]

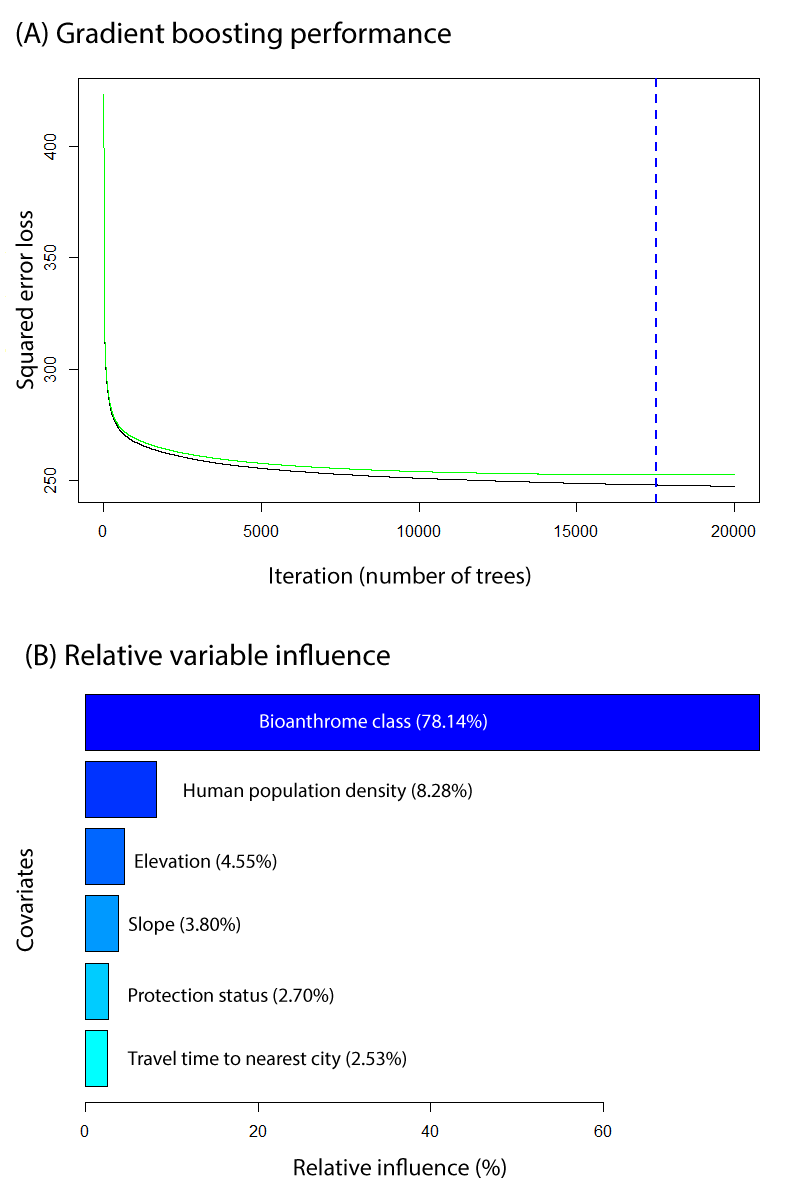

Supplement: Supplementary file 4 — Figure S4 [file COBI-39-e70092-s008.png]

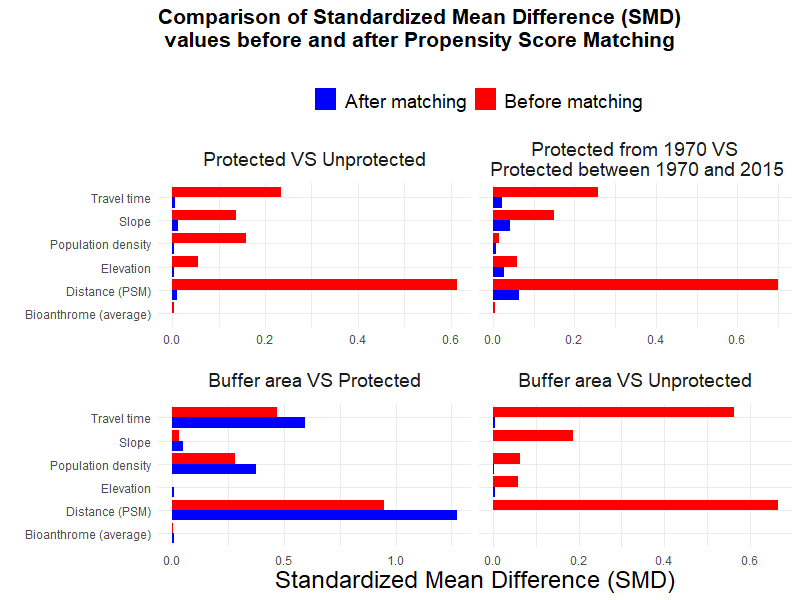

Supplement: Supplementary file 5 — Figure S5 [file COBI-39-e70092-s009.png]

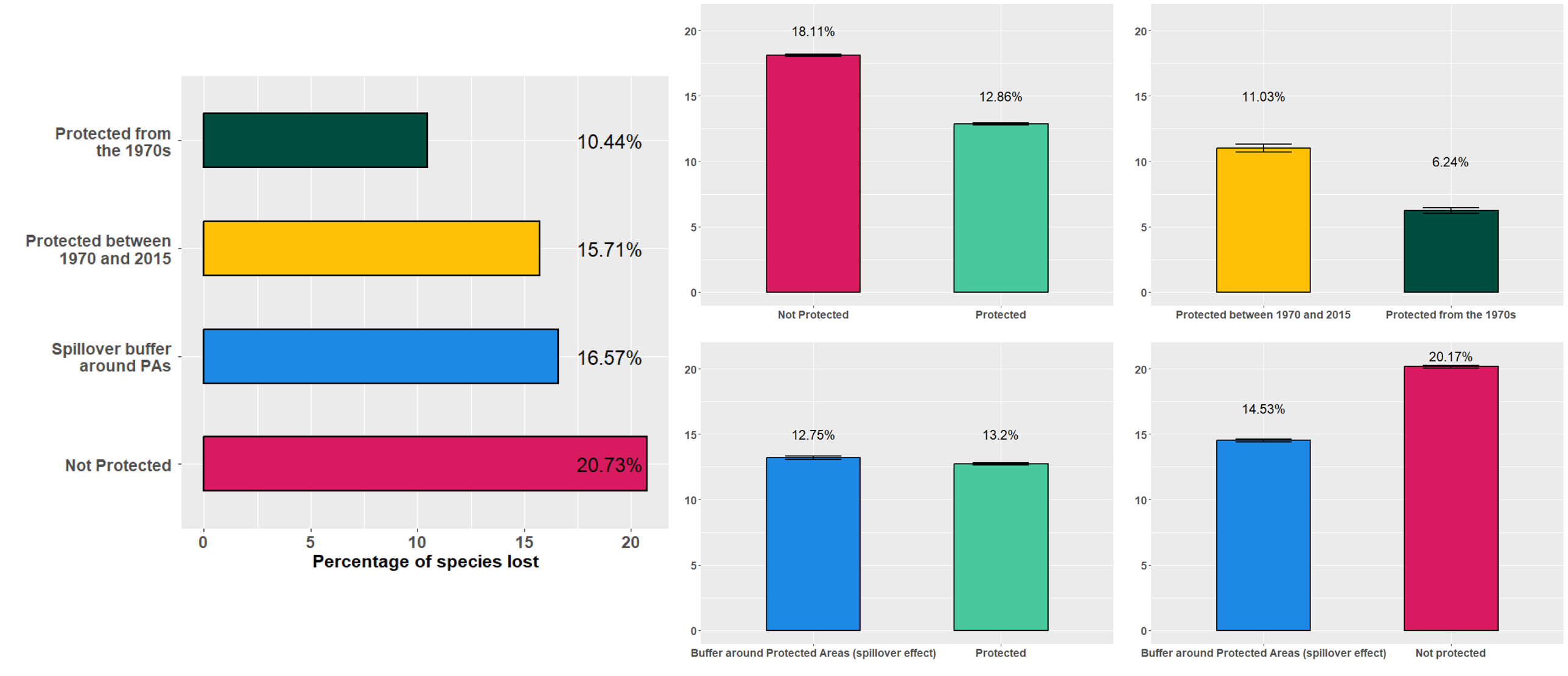

Supplement: Supplementary file 6 — Figure S6 [file COBI-39-e70092-s005.png]

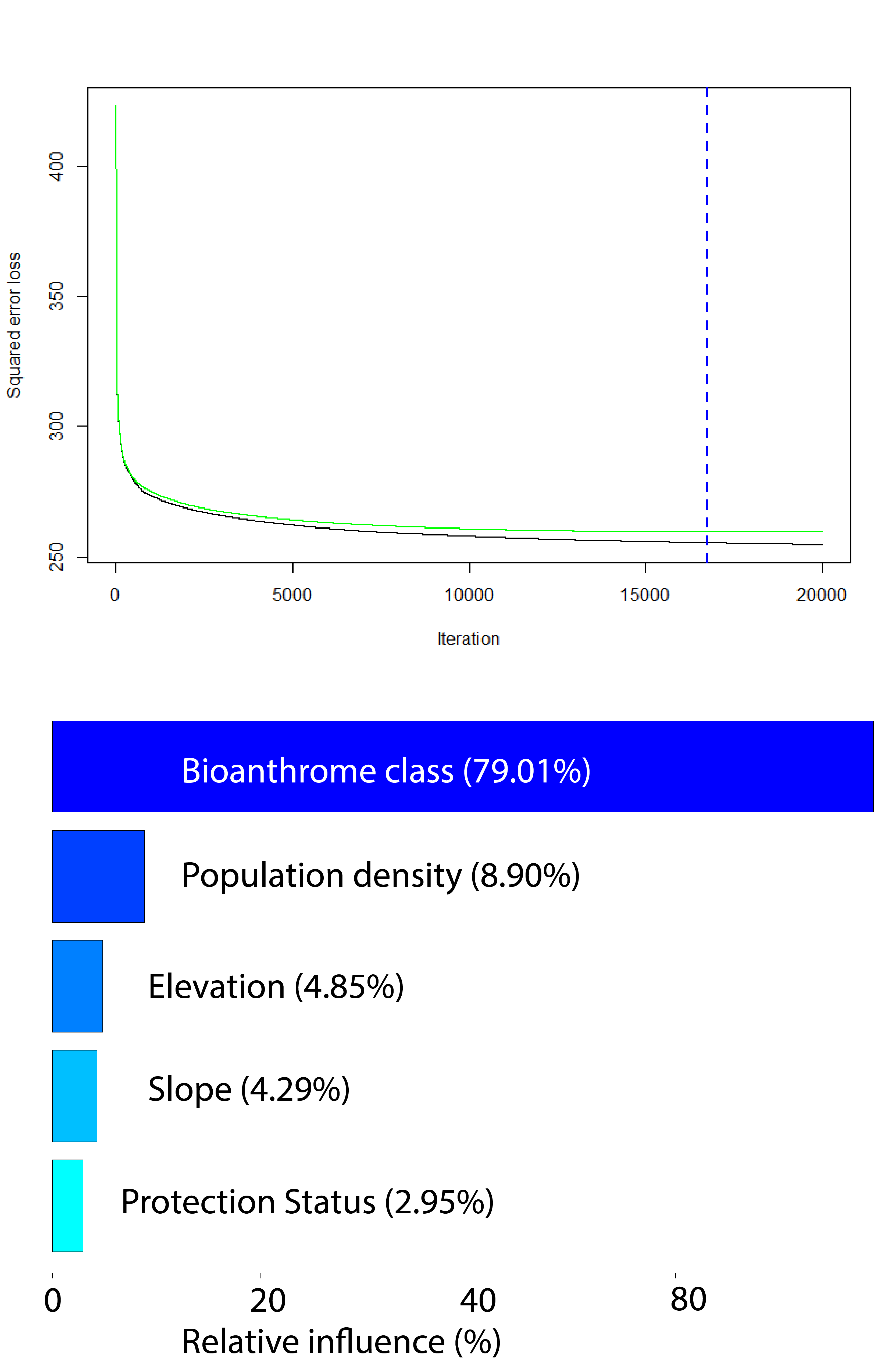

Supplement: Supplementary file 7 — Figure S7 [file COBI-39-e70092-s007.png]

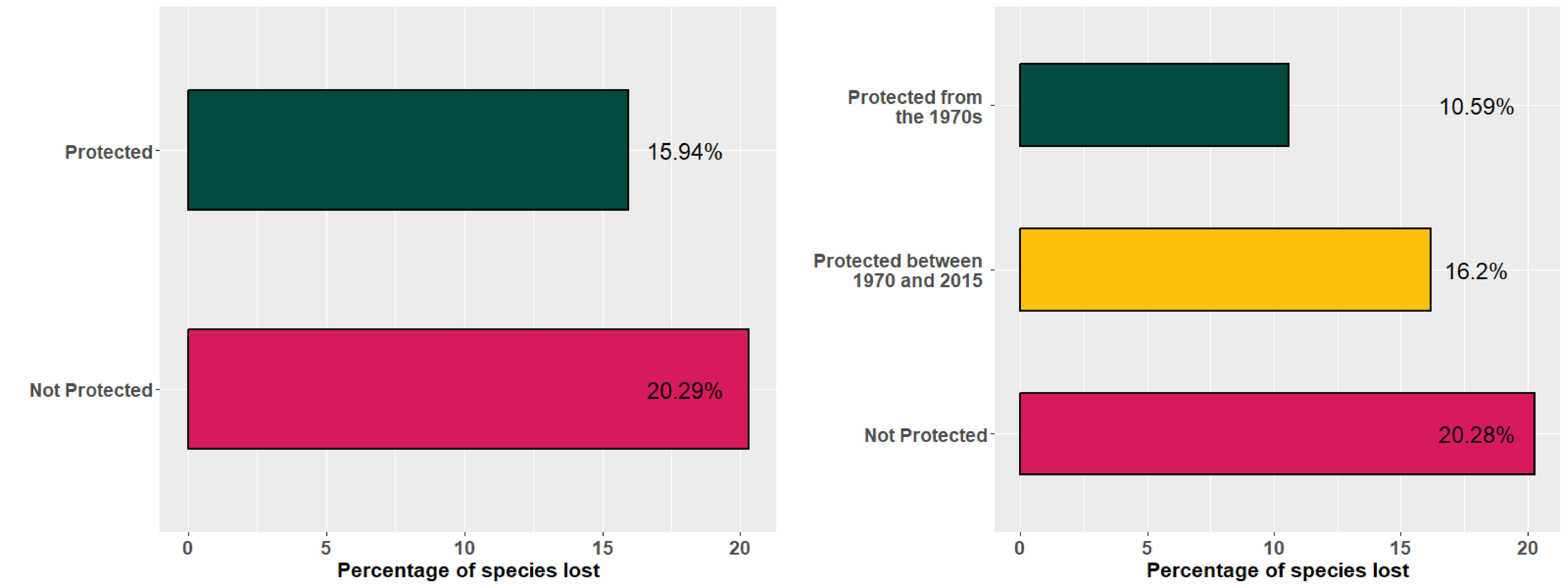

Supplement: Supplementary file 8 — Figure S8 [file COBI-39-e70092-s002.png]

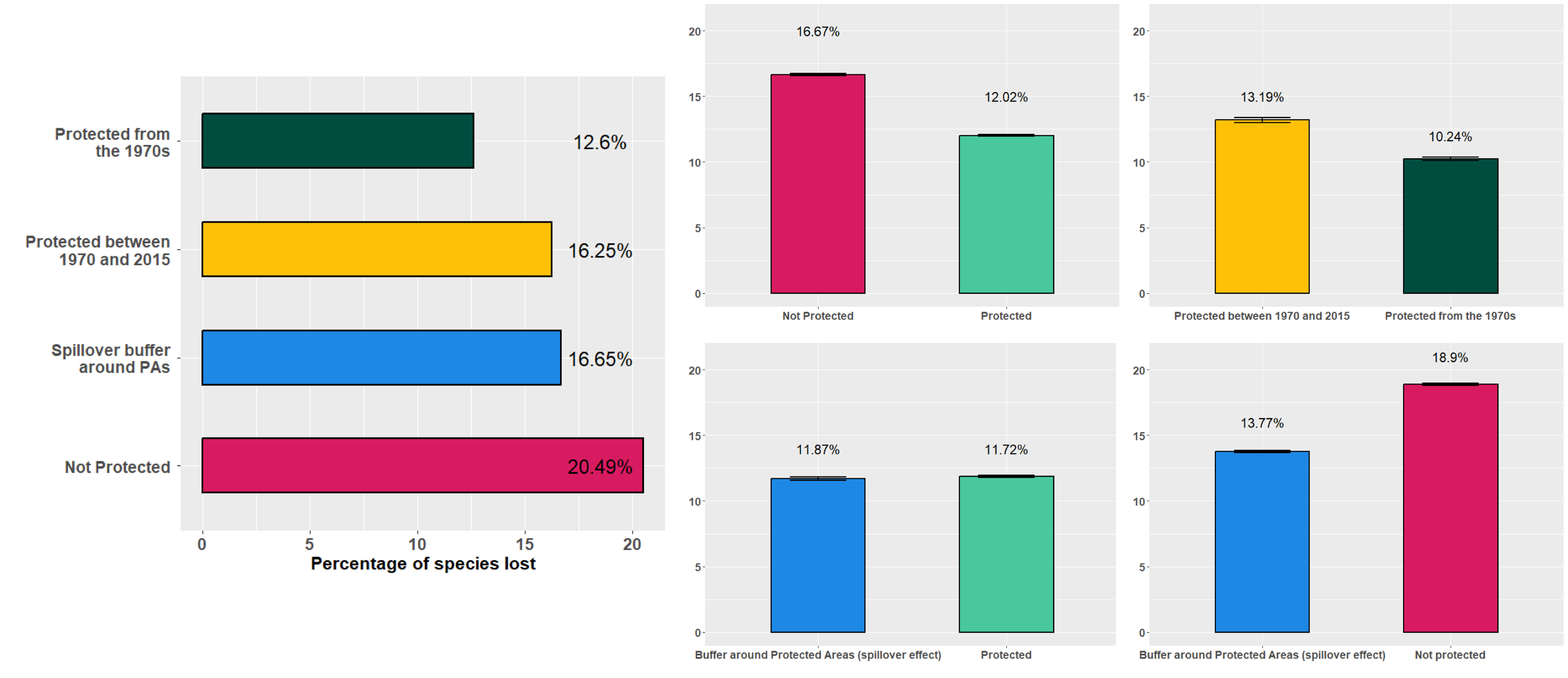

Supplement: Supplementary file 9 — Figure S9 [file COBI-39-e70092-s006.png]
