# Supplementary material for: Role of protected areas in mitigating range loss and local extinctions of terrestrial mammals
Source: Conserv Biol. 2025 Jun 16;39(6):e70092. doi: 10.1111/cobi.70092 (PMC12658939; doi:10.1111/cobi.70092)
Supplement: Supplementary file 10 — Supporting Information [file COBI-39-e70092-s004.docx]

**Supporting Information for:** The role of Protected Areas in mitigating range loss and local extinctions in terrestrial mammals

**Appendix S1. Collection and production of past range maps**

We further improved and updated the Pacifici et al. 2019 dataset with past distribution maps collected from the available peer-reviewed literature, including all research articles published between 1965 and 1989 that included a contemporary distribution range map. In doing so, we followed the procedures adopted in the original database, ensuring that the maps reported were consistent with descriptive accounts of species’ presence from the original articles and in the present literature (Pacifici et al., 2020). Imperfect knowledge meant that some of the past maps underestimated the true extent of the distribution ranges, missing sizable portions that are instead reported in the more recent IUCN assessments. In those cases where there was no reason to believe that genuine significant expansions occurred, and especially for threatened and declining species, we assumed that those missing portions were unreported in the past rather than representing true range increase, and therefore we included them as part of the past distributions. After checking for taxonomic changes to ensure that all the entities represented were the same for the past and the present, we aligned the taxonomy to the most recent one used by the IUCN Red List. Finally, we submitted the maps to more than 40 species and groups experts for the final steps of correction and validation. All distribution maps were developed and processed in WGS 1984 (EPSG:4326) coordinate reference system to maintain consistency with the Mapping Standards and Data Quality for IUCN Red List Spatial Data v1.19 (IUCN, 2021). The selection of 2015 as baseline year to represent the most recently known species’ range is also a consequence of the IUCN Red List assessments being seldom produced yearly, with the assessment process relying on information combined over months or years, which introduces small discrepancies in the temporal resolution of distribution maps (Pacifici et al., 2019, 2020).

IUCN Red List maps, which are the backbone of most conservation assessments and spatial planning, are produced by experts’ panels who integrate several sources, including field data, historical records, published literature and personal expertise, to delineate the approximate extent where a species can occur (Harfoot et al., 2021). Although these maps can be coarse, especially for species with very large ranges, they still provide a standardized and reproducible baseline to infer range extent (IUCN, 2021). At a resolution of 100 km^2^ , spatial averaging means that the granularity of the analysis is comparatively well matched to the inherent uncertainty of expert-drawn maps, making them suitable for broad-scale studies such as the one we presented in this manuscript. In addition, global and regional analyses of biodiversity are often conducted at similar relatively coarse spatial scales (Gaüzère et al. 2022; Hoffmann et al., 2008; Joppa et al. 2013; Jung et al. 2021; Visconti et al. 2016), and when the resolution of the analysis is broadly similar to or larger than the spatial uncertainty associated with range maps, the potential for misclassification of species presence/absence becomes greatly reduced (Joppa et al., 2016). All these considerations that apply to IUCN Red List maps for the current time period also apply to the past distribution maps that we created and validated with the supports of species’ experts involved in IUCN assessments, since they follow the same standardized methodology and guidelines.

**Appendix S2. Protection change between landscape units**

There was an increase in the proportional coverage of PAs across all bioanthromes, ranging from +0.3% (Irrigated Villages within Temperate Grasslands, Savannas & Shrublands) to +61.5% (Semi-natural treeless and barren lands within Flooded Grasslands & Savannas. In absence of protection, rainfed villages within temperate broadleaf and mixed forest were the bioanthromes with the highest local extinction rates (79.3%), while the lowest rates were registered for residential semi-natural woodlands within temperate grasslands (6.6%). Amongst landscape units that were protected continuously from the 1970s, populated rangelands within Mediterranean forest were amongst the bioanthromes with the highest extinctions rates (78.9%), while remote semi-natural woodlands within flooded grasslands and savannas had the lowest rates (4.4%).

In the 1970s biomes were protected on average only on 0.6% of their extent, and in 2015 the proportion of protection increased to 12.7%. Tropical & Subtropical Coniferous Forests were the only biome without any PAs in the 1970s, but became protected on 12.7% of their extent in 2015. All biomes experienced an increase in the proportion of their extent being encompassed within PAs, ranging from a minimum + 3.2% for Temperate Grasslands, Savannas & Shrublands to a maximum +28.6% for Flooded Grasslands & Savannas. Local extinction rates were 30.7% averaged across all biomes, but there were substantial differences between categories of protection status. With the exception of Temperate Broadleaf & Mixed Forests, rates of extinctions in biomes protected since the 1970s were considerably lower compared to biomes that were totally unprotected, with the 2015 timestep often providing an intermediate estimate. Flooded Grasslands & Savannas represented the biome with the largest decrease in biodiversity loss between areas unprotected (33.1% species lost) and areas protected from the 1970s (13.2% species lost), while the smallest difference was observed in Temperate Grasslands, Savannas & Shrubland (34.3% loss in unprotected, 29.1% in Protected from 1970). Interestingly, those biomes had respectively the largest and smallest increase in the proportions of their protected extents.

As for anthromes, they were protected on average over 0.4% of their extent in 1970, and on 9.9% in 2015. Like biomes, all anthromes showed an increase in protection, ranging from a minimum +1.2% for the category of rice-cultivating villages to a maximum of +31.6% for the category of wild, remote icy regions. Rates of species loss averaged over all anthromes were 36.9%, again with noticeable differences between categories of protection status. Almost all anthromes protected since the 1970s showed lower rates of extinctions compared to the same anthromes that were never protected, albeit the differences being less marked compared to those observed for biomes. Notable exceptions were populated rangelands, and residential and populated semi-natural woodlands. Similarly to biomes, the 2015 timestep in protection was often an intermediate estimate between the 1970s timestep and the absence of protection. Rice-cultivating villages were the anthrome with the largest differences in rates of biodiversity loss between unprotected (48%) and protected from the 1970s (14.9%), despite also being the anthrome with the smallest proportional expansion of PAs. The anthromes showing the smallest decrease in biodiversity loss between unprotected and protected from the 1970s were dense settlements (-2.8%).

**Appendix S3. Associations with ecological and life-history traits**

We also evaluated whether rates of range loss and change in proportional area of protected range were associated with mammal species’ ecological and life-history traits usually employed in assessments of species’ status. We extracted a total of 17 quantitative traits from COMBINE, a coalesced database of 54 intrinsic and extrinsic traits collected and/or imputed for 6,234 mammal species (Soria et al., 2021). From the database, we extracted quantitative measurements of adult body mass (in grams), adult body length (in millimetres), maximum longevity (in days), age of maturity (in days), age at first reproduction (in days), gestation length (in days), litter size (mean number of litter produced in a single reproductive event), reproductive rates (number of reproductive events in a year), interbirth interval (in days), generation length (in days), dispersal distance (in kilometres), population density (number of individuals per square kilometre), home range size (in square kilometres), social group size (number of individuals in social group), upper and lower elevational limits of the distribution range (in meters), and altitude breadth between lower and upper elevational limits (in metres). We used all these traits as predictor variables in two generalized linear models, using two linear regressions formulated as:

$range lost \left( \text{\%} \right) \sim body mass \left( g \right)+body length \left( mm \right)+longevity \left( days \right)+maturity \left( days \right)+age at first reproduction \left( days \right)+gestation length \left( days \right)+litter size+litter per year+interbirth interval \left( days \right)+generation length \left( days \right)+dispersal \left( km \right)+population density \left( \frac{n}{km2} \right)+home range \left( km2 \right)+upper elevation limit \left( m \right)+lower elevation limit (m)$ (S1), and

$change in PA coverage \left( \text{\%} \right) \sim body mass \left( g \right)+body length \left( mm \right)+longevity \left( days \right)+maturity \left( days \right)+age at first reproduction \left( days \right)+gestation length \left( days \right)+litter size+litter per year+interbirth interval \left( days \right)+generation length \left( days \right)+dispersal \left( km \right)+population density \left( \frac{n}{km2} \right)+home range \left( km2 \right)+upper elevation limit \left( m \right)+lower elevation limit (m)$ (S2),

where the percentage of species locally extinct (equation S1) and the change in the proportion of protected range (equation S2) were both modelled as additive functions of species’ ecological and life-history traits. We did not find any kind of significant correlation between traits and range change (Appendix S2), nor between traits and change in protection coverage (Appendix S3), underlining that for the mammal species of our sample, range contractions and increase of protected areas seem to have occurred indiscriminately.

**Appendix S4. Technical details on rasterization of vector data**

Data collected in shapefile vectors format (WDPA data, species range maps, and biome boundaries) was converted in raster grids using the default behaviour of the Rasterize (Vector to Raster) function implemented in QGIS, which is based on the gdal_rasterize function implemented within GDAL - the Geospatial Data Abstraction Library that QGIS is based on for most of its function. By default, the QGIS Rasterize function operates as a two-step algorithm. In the first step, all the pixel at a specified resolution that are completely outside the polygon to be rasterized are removed. In the second step, all the pixels that overlap with the boundaries of the polygon, and that have their centre point located outside of the polygon boundaries, are also removed. This implementation of the Rasterize function tends to avoid overestimation of valid pixels distributions. In our case, all the cells overlapping with shapefile boundaries but with their centre point located outside those boundaries, were not considered to be protected. More specifically, since we used a 10 kilometre buffer to assess spillover effects, all the aforementioned cells ended being included in the spillover buffer around PA boundaries (and consequently all the pixel cells located outside the buffer zones were considered completely unprotected).

**Appendix S5. Robustness checks**

We performed a series of robustness check to ensure the consistency of our results, and to ensure that our findings were not sensitive to the methodological and analytical choices of our models.

1. **Inclusion and exclusion of time travel to cities from covariates**

In our assessment of the effectiveness of PAs at mitigating declines for mammal species, we used a set of covariates that were either time-invariant, such as elevation and slope, or quantified pre-treatment, i.e. for the baseline year 1970, such as human population density and bioanthrome classification. However, data for travel time to the nearest city was not available before the year 2000, and as such it was not quantified for the baseline year 1970 as true pre-treatment. To ensure that the inclusion of this covariate did not significantly affect our results, we ran an additional version of the original analysis, dropping time travel to cities from the list of covariates explaining species loss in the Generalized Boosted Models and Propensity Score Matchings. Results of this robustness check are presented in Appendix S11 and S12, and show that the outputs of both GBMs and PSMs do not change regardless of the inclusion or exclusion of time travel for the year 2000 in the covariates.

1. **Exclusion of potential spillover areas from the control regions**

In our assessment of the effectiveness of PAs at mitigating declines for mammal species, we considered pixels of 100 km^2^ adjacent to the boundaries of Protected Areas as regions where potential spillover effects could occur. We conducted additional robustness checks removing the 10 km buffer around Protected Areas from the control, to ensure that our results were not contaminated by potential spillover. For Propensity Score Matching, this analysis is already integrated within the manuscript as the pairwise comparison between all protected landscape units and all unprotected landscape units, with pixels in the buffer zone around PAs excluded from the control (see Figure 2B). For Generalized Boosted Models, we ran a supplementary analysis twice, once with all Protected Areas merged together and compared against unprotected areas, and a second time with PAs categorized based on the time of their establishment (either before or after 1970, as in the main GBM analysis). We found that the results of these robustness check are very consistent with those of our primary analysis (see Appendix S13), with rates of species loss in protected and unprotected units not being substantially affected by the presence or absence of buffer areas where spillover may occur.

1. **Exclusion of Protected Areas with undefined boundaries**

In our assessment of the effectiveness of PAs at mitigating declines for mammal species, we also checked whether the outcome of our analyses was sensitive to the inclusion of data for spatially ill-defined PAs, and more specifically all those PAs for which there was no polygon with a defined boundary but only a centroid point associated with the extent of the PAs. We conducted this robustness check by running all of our analyses again, this time only including PAs with boundaries spatially defined in the WDPA database and removing PAs for which the boundaries were approximated as a circular area around a centroid point. Similar to the primary analysis of the manuscript, we ran a GBM comparing all the level categories of protection status, and four PSM with pairwise comparison between level categories of protection status. We found that the results of this last robustness check are also consistent with those of our primary analyses (see Appendix S14), showing that the outcome of both PSM and GBMs do not change regardless of the inclusion or exclusion from the dataset of ill-defined PAs with a circular buffer area built around a centroid point.

**Supporting Information references**

Gaüzère, P., O’connor, L., Botella, C., Poggiato, G., Münkemüller, T., Pollock, L. J., ... & Thuiller, W. (2022). The diversity of biotic interactions complements functional and phylogenetic facets of biodiversity. *Current Biology*, *32*(9), 2093-2100.

Harfoot, M. B., Johnson, A., Balmford, A., Burgess, N. D., Butchart, S. H., Dias, M. P, Hazin, C., Hilton-Taylo, C., Hoffmann, M., Isaac, N. J. B., Iversen, L. L., Outhwaite, C., Visconti, P., & Geldmann, J. (2021). Using the IUCN Red List to map threats to terrestrial vertebrates at global scale. *Nature Ecology & Evolution, 5*(11), 1510-1519

Hoffmann, M., Brooks, T. M., Da Fonseca, G. A. B., Gascon, C., Hawkins, A. F. A., James, R. E., Langhammer, P., Mittermeier, R. A., Pilgrim, J. D., Rodrigues, A. S. L., & Silva, J. M. C. (2008). Conservation planning and the IUNC Red List. *Endangered Species Research 6*(2), 113-125

IUCN (2021). Mapping Standard and Data Quality for IUCN Red List Spatial Data. <https://iucnredlist.org/resources/mappingstandards>. Accessed on 05/06/2024

Joppa, L. C., Visconti, P., Jenkins, C. N. & Pimm, S. L. (2013). Achieving the convention on biological diversity’s goals for plant conservation. *Science, 341*(6150), 1100-1103.

Joppa, L. C., Butchart, S. H. M., Hoffmann, M., Bachman, S. P., Resit Akçakaya, H., Moat, J. F., Bohm, M., Holland, R. A., Newton, A., Polidoro, B., & Hughes, A. (2016). Impact of alternative metrics on estimates of extent of occurrence for extinction risk assessment. *Conservation Biology, 30*(2), 362-370

Jung, M., Arnell, A., De Lamo, X., García-Rangel, S., Lewis, M., Mark, J., ... & Visconti, P. (2021). Areas of global importance for conserving terrestrial biodiversity, carbon and water. *Nature Ecology & Evolution*, *5*(11), 1499-1509.

Pacifici, M., Cristiano, A., Burbidge, A. A., Woinarski, J. C. Z., Di Marco, M., & Rondinini, C. (2019). Geographic distribution ranges of terrestrial mammal species in the 1970s. Ecology, 100(7). https://doi.org/10.1002/ecy.2747

Pacifici, M., Rondinini, C., Rhodes, J. R., Burbidge, A. A., Cristiano, A., Watson, J. E. M., Woinarski, C., & Di Marco, M. (2020). Global correlates of range contractions and expansions in terrestrial mammals. *Nature Communications, 11*(1), 2840.

Soria, C. D., Pacifici, M., Di Marco, M., Stephen, S. M., & Rondinini, C. (2021). COMBINE: a coalesced mammal database of intrinsic and extrinsic traits. *Ecology*, *102*(6). <https://doi.org/10.1002/ecy.3344>

Visconti, P., Bakkenes, M., Baisero, D., Brooks, T., Butchart, S. H., Joppa, L., ... & Rondinini, C. (2016). Projecting global biodiversity indicators under future development scenarios. *Conservation Letters*, *9*(1), 5-13.

**Supporting Information tables**

**Appendix S6 (sample taxonomic coverage).**

| **Taxonomic  Order** | **Number of  sampled species** | **Number of  known species** | **Percentage of  species represented** |
| --- | --- | --- | --- |
| *Afrosoricida* | 4 | 55 | 7.3% |
| *Carnivora ** | 99 | 261 | 37.9% |
| *Cetartiodactyla ** | 133 | 243 | 54.7% |
| *Chiroptera* | 26 | 1,332 | 1.9% |
| *Cingulata* | 2 | 20 | 10% |
| *Dasyuromorphia* | 6 | 72 | 8.3% |
| *Dermoptera* | 0 | 2 | 0% |
| *Didelphimorphia* | 2 | 98 | 2% |
| *Diprotodontia* | 7 | 147 | 4.8% |
| *Eulipotyphla* | 9 | 494 | 1.8% |
| *Hyracoidea* | 4 | 5 | 80% |
| *Lagomorpha* | 16 | 96 | 16.7% |
| *Macroscelidea* | 16 | 19 | 84.2% |
| *Microbiotheria* | 1 | 1 | 100% |
| *Monotremata* | 0 | 5 | 0% |
| *Notoryctemorphia* | 0 | 2 | 0% |
| *Paucitubercolata* | 0 | 7 | 0% |
| *Peramelemorhphia* | 2 | 22 | 9.1% |
| *Perissodactyla* | 10 | 16 | 62.5% |
| *Pholidota* | 4 | 8 | 50% |
| *Pilosa* | 0 | 10 | 0% |
| *Primates* | 67 | 522 | 12.8% |
| *Proboscidea* | 2 | 3 | 66.7% |
| *Rodentia* | 72 | 2,375 | 3% |
| *Scandentia* | 0 | 23 | 0% |
| *Tubulidentata* | 1 | 1 | 100% |
| **Total** | **483** | **5,839** | **8.3%** |

**Appendix S6.** Number of mammal species included in our sample and percentages of known terrestrial mammal species for each Order included in our sample. Average taxonomic representation of mammal species equals 8.3%. For Orders marked with an asterisk (*), the number of known species refers exclusively to terrestrial species.

**Appendix S7 (range contraction associations with change in Protected range and extent of Protection).**

| **Model formula** | **Percentage of range lost ~  proportional change of PA coverage** | | | **Percentage of range lost ~  range area protected in 1970s** | | | **Percentage of range lost ~  range area protected in 2015** | | |
| --- | --- | --- | --- | --- | --- | --- | --- | --- | --- |
| *Predictors* | *Estimates* | *95%  Conf. Int.* | *p-value* | *Estimates* | *95%  Conf. Int.* | *p-value* | *Estimates* | *95%  Conf. Int.* | *p-value* |
| Intercept | 0.343 | 0.317 –  0.369 | **< 0.001 ***** | 0.478 | 0.410 –  0.547 | **< 0.001 ***** | 0.758 | 0.670 –  0.845 | **< 0.001 ***** |
| Proportional change of  PA range coverage | -0.039 | -0.051 –  -0.028 | **< 0.001 ***** | - | - | - | - | - | - |
| Range area protected  in 1970 (log km^2^) | - | - | - | -0.017 | -0.024 –  -0.010 | **< 0.001 ***** | - | - | - |
| Range area protected  in 2015 (log km^2^) | - | - | - | - | - | - | -0.040 | -0.048 –  -0.033 | **< 0.001 ***** |
| Multiple R^2^ / Adjusted R^2^ | 0.087 / 0.085 | | | 0.048 / 0.046 | | | 0.178 / 0.176 | | |
| Residual standard error | 0.2776 on 481  degrees of freedom | | | 0.2835 on 481  degrees of freedom | | | 0.2635 on 481  degrees of freedom | | |
| F-statistic | 45.98 on 1 and 481  degrees of freedom | | | 24.15 on 1 and 481  degrees of freedom | | | 104 on 1 and 481  degrees of freedom | | |
| Model p-value | **< 0.001 ***** | | | **< 0.001 ***** | | | **< 0.001 ***** | | |

**Appendix S7.** Summary of the generalized linear models estimating species’ range contraction as a function of changes in proportional PA coverage over species ranges, and as a function of the natural logarithm of the extent of Protected ranges in 1970 and 2015. All of the predictors are significant in explaining range contraction across all three models.

**Appendix S8 (species loss rates associations with change in bioanthrome Protection coverage and bioanthrome area).**

| **Model formula** | **Rates of species loss ~ change of bioanthrome PA coverage + bioanthrome area (km^2^)** | | | **Rates of species loss ~ change of bioanthrome PA coverage + bioanthrome area (log km^2^)** | | |
| --- | --- | --- | --- | --- | --- | --- |
| *Predictors* | *Estimates* | *95% Confidence Interval* | *p-value* | *Estimates* | *95% Confidence Interval* | *p-value* |
| Intercept | 35.701 | 33.504 – 37.898 | **< 0.001 ***** | 35.059 | 26.035 – 44.083 | **< 0.001 ***** |
| Percentage change  of PA coverage over bioanthromes | -0.252 | -0.369 – -0.135 | **< 0.001 ***** | -0.251 | -0.369 – -0.133 | **< 0.001 ***** |
| Bioanthrome area (km^2^) | 0.000 | 0.000 – 0.000 | 0.995 | - | - | - |
| Bioanthrome area (log km^2^) | - | - | - | 0.059 | -0.746 – 0.864 | 0.885 |
| Multiple R^2^ / Adjusted R^2^ | 0.065 / 0.058 | | | 0.065 / 0.054 | | |
| Residual standard error | 12.51 on 262 degrees of freedom | | | 12.51 on 262 degrees of freedom | | |
| F-statistic | 9.106 on 2 and 262 degrees of freedom | | | 9.117 on 2 and 262 degrees of freedom | | |
| Model p-value | **< 0.001 ***** | | | **< 0.001 ***** | | |

**Appendix S8.** Summary of the generalized linear models estimating species’ range contraction as a function of changes in proportional PA coverage over bioanthrome and bioanthrome size. Bioanthrome size is used either as area in km^2^ or in its natural logarithm in two different models. Only the change in Protection coverage is a significant predictor in both models.

**Appendix S9 (range contraction associations with ecological and life-history traits).**

| **Model formula** | **Percentage of range lost ~ species traits** | | |
| --- | --- | --- | --- |
| *Predictors* | *Estimates* | *95% Confidence Interval* | *p-value* |
| Intercept | 1.09347 | 0.33019 – 1.85675 | **0.007 **** |
| Adult body mass (g) | 0.00000 | -0.00000 – 0.00000 | 0.399 |
| Adult body length (mm) | -0.00021 | -0.00051 – 0.00010 | 0.174 |
| Max longevity (days) | -0.00007 | -0.00016 – 0.00003 | 0.169 |
| Maturity (days) | -0.00008 | -0.00061 – 0.00045 | 0.749 |
| Age at first reproduction (days) | 0.00025 | -0.00019 – 0.00068 | 0.251 |
| Gestation length (days) | 0.00105 | -0.00122 – 0.00332 | 0.346 |
| Litter size (numerical) | -0.08127 | -0.19345 – 0.03091 | 0.147 |
| Litter per year (numerical) | -0.15290 | -0.42090 – 0.11509 | 0.249 |
| Interbirth interval (days) | 0.00003 | -0.00082 – 0.00087 | 0.945 |
| Generation length (days) | -0.00000 | -0.00033 – 0.00033 | 0.989 |
| Dispersal (km) | 0.00314 | -0.02056 – 0.02685 | 0.785 |
| Density (n/km^2^) | 0.00025 | -0.00078 – 0.00128 | 0.618 |
| Home range (km^2^) | -0.00005 | -0.00080 – 0.00070 | 0.893 |
| Social group (numerical) | 0.00321 | -0.00598 – 0.01240 | 0.475 |
| Upper elevation (m) | -0.00003 | -0.00014 – 0.00007 | 0.525 |
| Lower elevation (m) | -0.00018 | -0.00056 – 0.00020 | 0.333 |
| Multiple R^2^ / Adjusted R^2^ | 0.513 / 0.143 | | |
| Residual standard error | 0.2618 on 21 degrees of freedom | | |
| F-statistic | 1.385 on 16 and 21 degrees of freedom | | |
| Model p-value | 0.2389 | | |

**Appendix S9.** Summary of the generalized linear model estimating species’ range change as a function of species ecological and life history traits. There are no significant associations between range change and species traits.

**Appendix S10 (change in protection coverage associations with ecological and life-history traits).**

| **Model formula** | **Change in the proportion of protected range ~ species traits** | | |
| --- | --- | --- | --- |
| *Predictors* | *Estimates* | *95% Confidence Interval* | *p-value* |
| Intercept | 0.35389 | 0.04236 – 0.66543 | **0.028 *** |
| Adult body mass (g) | -0.00000 | -0.00000 – 0.00000 | 0.913 |
| Adult body length (mm) | -0.00010 | -0.00022 – 0.00003 | 0.116 |
| Max longevity (days) | -0.00003 | -0.00007 – 0.00001 | 0.148 |
| Maturity (days) | -0.00010 | -0.00032 – 0.00011 | 0.339 |
| Age at first reproduction (days) | 0.00014 | -0.00004 – 0.00032 | 0.117 |
| Gestation length (days) | 0.00002 | -0.00090 – 0.00095 | 0.964 |
| Litter size (numerical) | -0.01135 | -0.05714 – 0.03444 | 0.612 |
| Litter per year (numerical) | -0.06948 | -0.17886 – 0.03990 | 0.201 |
| Interbirth interval (days) | -0.00014 | -0.00048 – 0.00021 | 0.419 |
| Generation length (days) | 0.00006 | -0.00007 – 0.00020 | 0.337 |
| Dispersal (km) | 0.00566 | -0.00401 – 0.01534 | 0.237 |
| Density (n/km^2^) | 0.00001 | -0.00041 – 0.00043 | 0.960 |
| Home range (km^2^) | -0.00005 | -0.00036 – 0.00026 | 0.743 |
| Social group (numerical) | 0.00148 | -0.00227 – 0.00523 | 0.422 |
| Upper elevation (m) | 0.00000 | -0.00004 – 0.00004 | 0.905 |
| Lower elevation (m) | -0.00009 | -0.00024 – 0.00007 | 0.250 |
| Multiple R^2^ / Adjusted R^2^ | 0.373 / - 0.105 | | |
| Residual standard error | 0.1068 on 21 degrees of freedom | | |
| F-statistic | 0.7796 on 16 and 21 degrees of freedom | | |
| Model p-value | 0.691 | | |

**Appendix S10.** Summary of the generalized linear model estimating the change in proportional coverage of PA over species’ ranges as a function of species ecological and life history traits. There are no significant associations between change in PA coverage and species traits.

**Supporting Information figures**

**
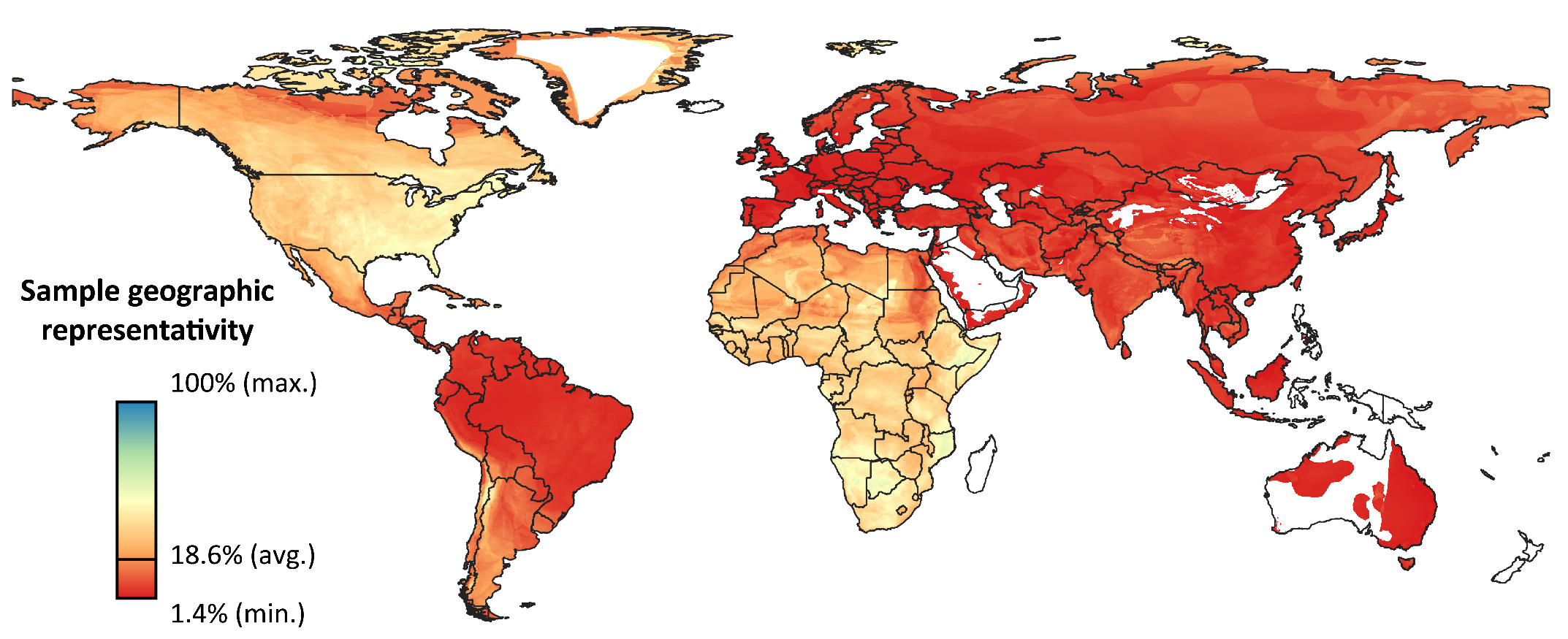
**

**Appendix S11.** Geographic representation of species included in the dataset (n = 483 species), expressed as the percentage of sampled mammal species over currently existing mammal species for each pixel. Taxonomic representation was calculated as the ratio of the sampled species co-occurring in the recent time period to the number of extant mammal species known to occur within each pixel. Scale resolution = 0.0833 degrees = 5 arc minutes = approximately 10 km at the Equator (pixel area is approximately 100 km^2^).

**
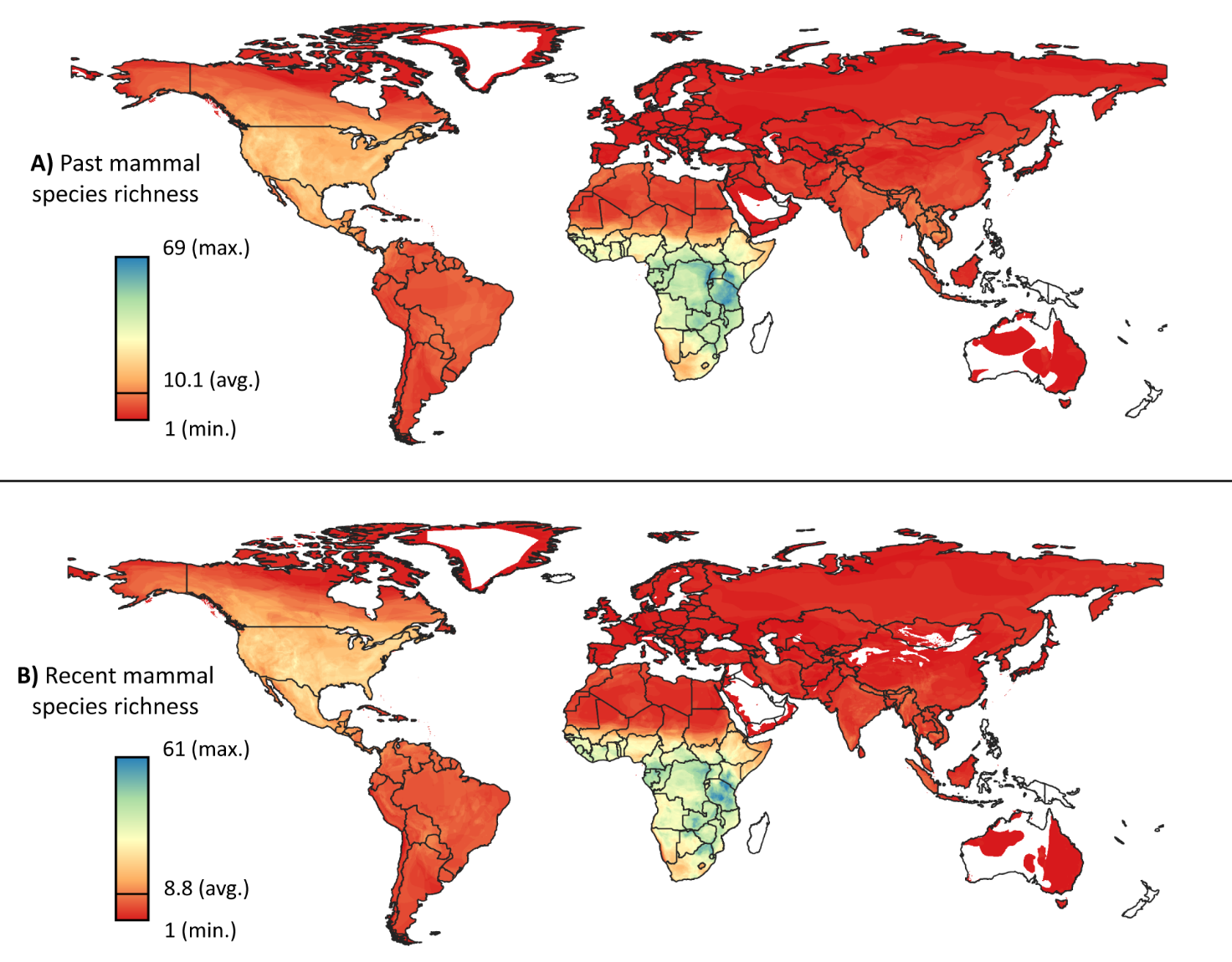
**

**Appendix S12.** Richness of the sampled species of mammals (n = 483) for the past (1970s) and recent (2015) time periods, calculated as the sum of mammal species co-occurring within each pixel. Maximum and average species richness were higher in the 1970s (A) compared to 2015 (B). Scale resolution = 0.0833 degrees = 5 arc minutes = approximately 10 km at the Equator (pixel area is approximately 100 km^2^).

**
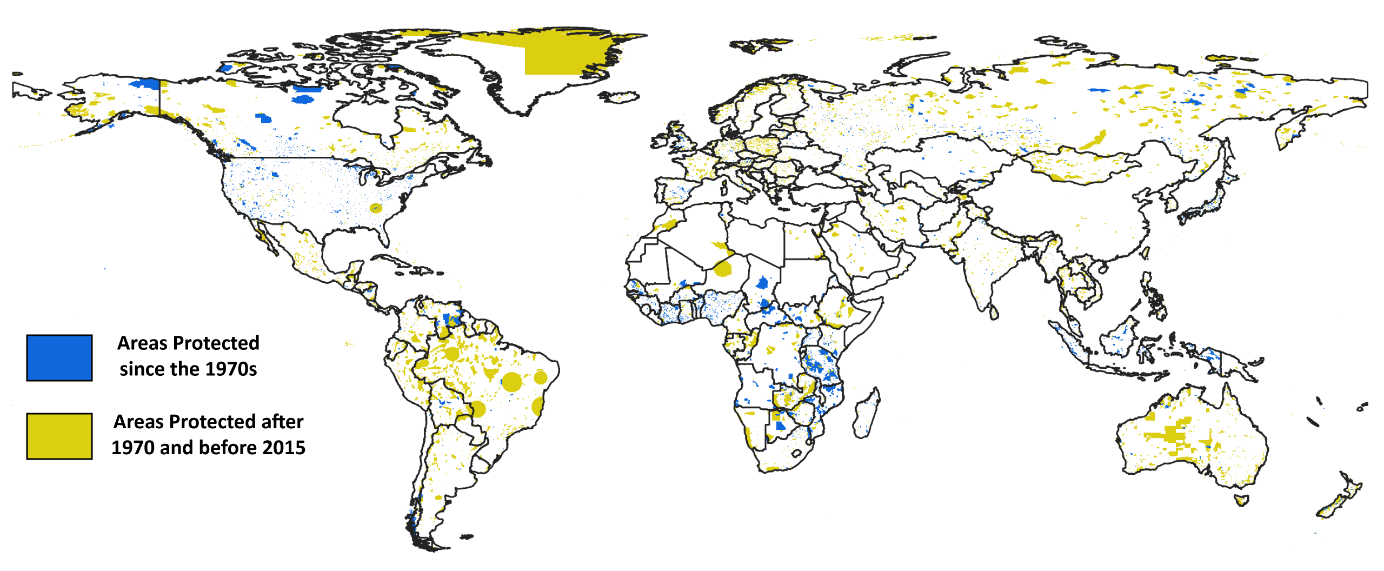
**

**Appendix S13.** Location and extent of terrestrial Protected Areas between 1970 and 2015. Areas that have been protected continuously since the 1970s (i.e. PAs established before 1970) are represented in blue. Protected Areas established after 1970 and before 2015 are represented in yellow. Areas in white are not and have never been Protected. Scale resolution = 0.0833 degrees = 5 arc minutes = approximately 10 km at the Equator (pixel area is approximately 100 km^2^).

**
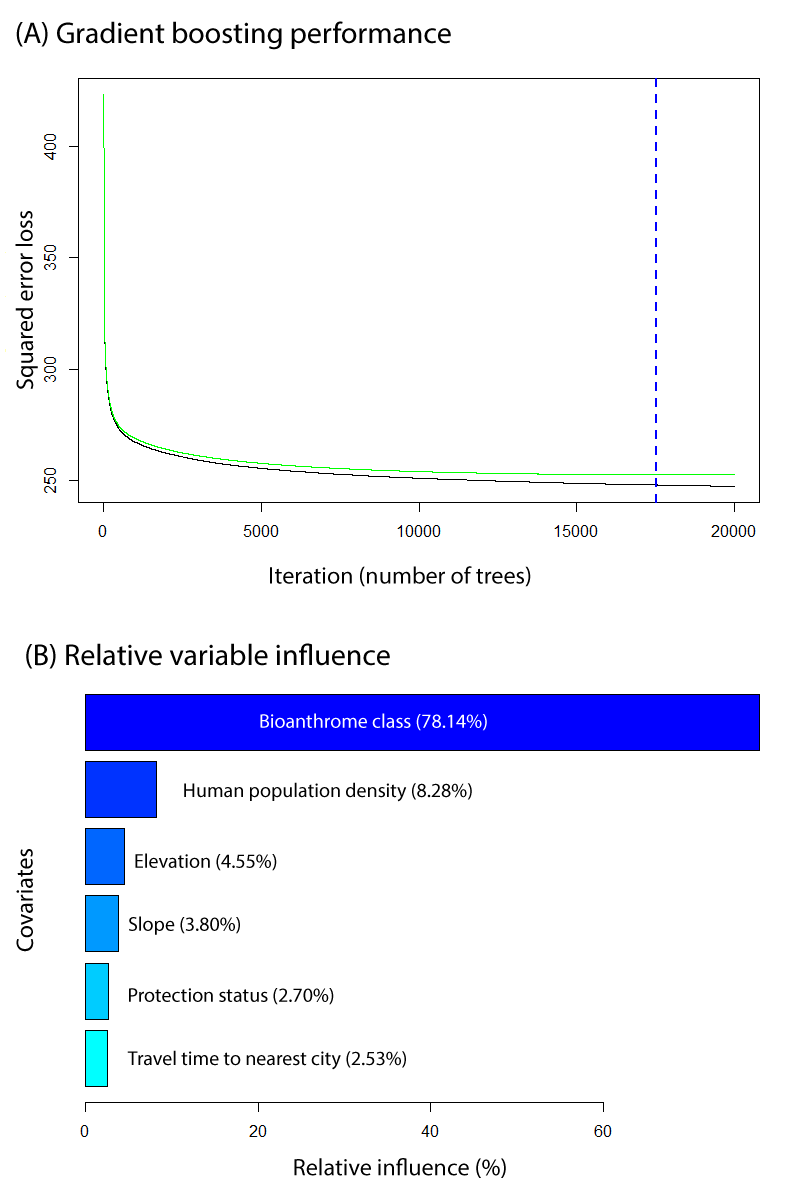
**

**Appendix S14.** Performance of Generalized Boosted Model with 20,000 trees. Loss function as a result of the number of trees added to the ensemble shows that performance does not increase more than a factor of 0.1 at around 17,500 trees (A). After fitting the model, bioanthrome classification is the covariate with the highest relative influence in decreasing the mean squared error loss function (B), highlighting a large degree of differentiation of PAs performance across bioanthromes.


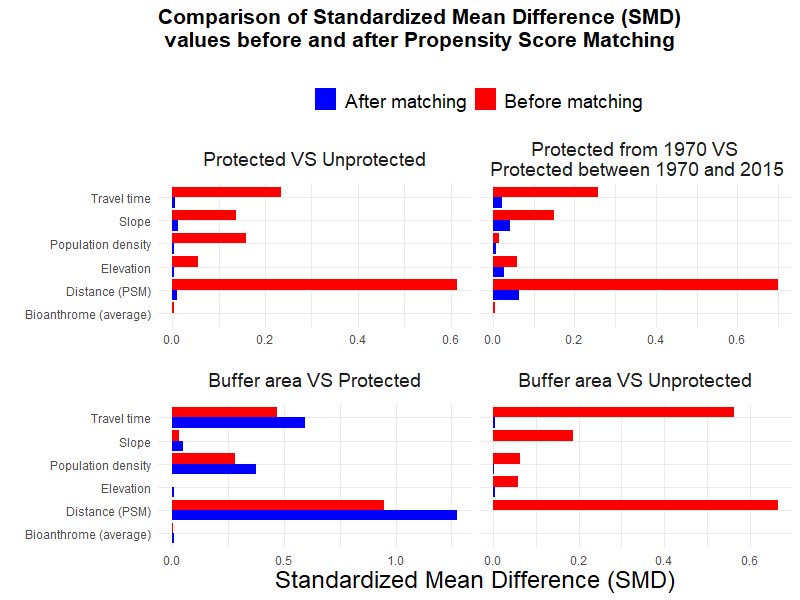


**Appendix S15.** Values of Standardized Mean Difference of continuous covariates before and after matching, for each of the pairwise comparison assessed with Propensity Score Matching. Only continuous variables and the average of bioanthrome classes are shown in the figure.


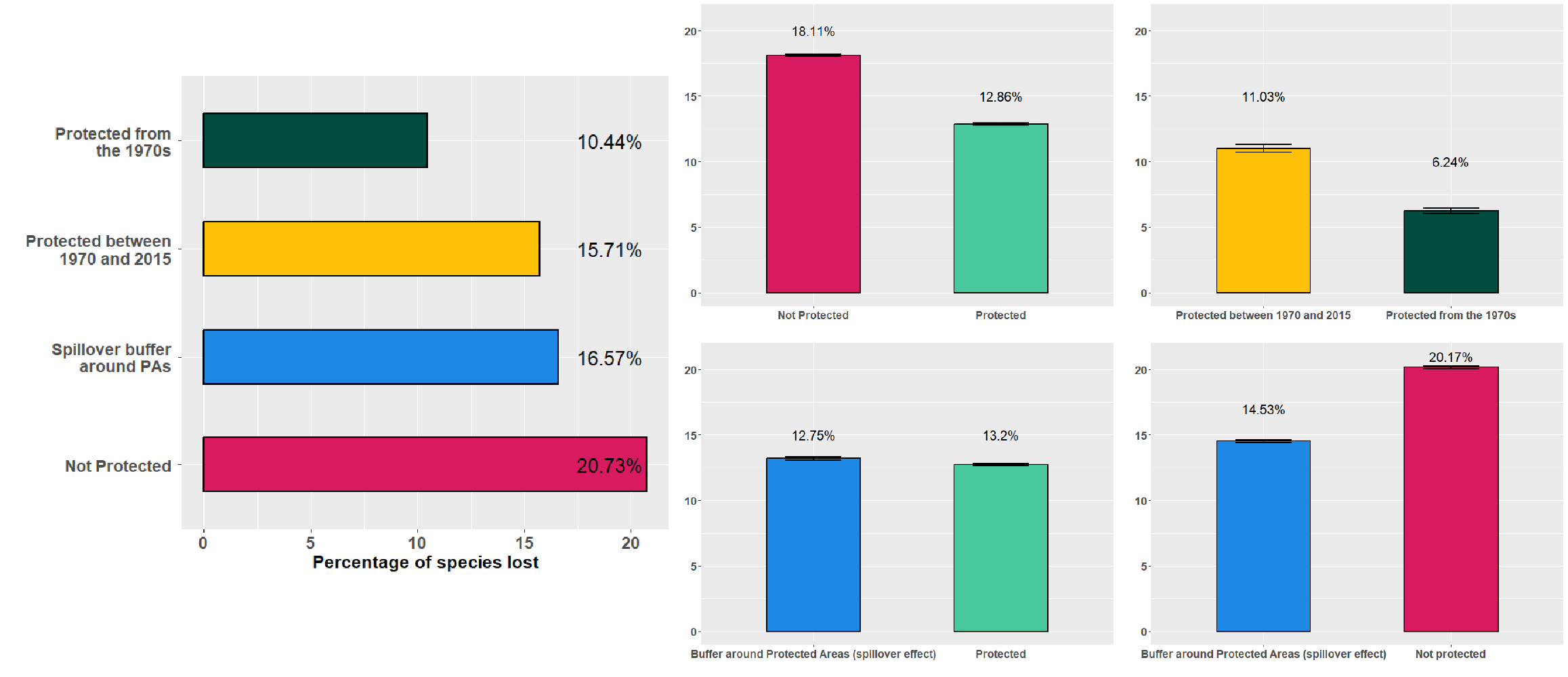


**Appendix S16.** Differences in rates of species loss across matched landscape units with different protection status, based on Generalized Boosted Models and Propensity Score Matching with no travel time to cities as covariate explaining species loss. Compare to Figure 2 for reference to the outcome of the primary analysis. The inclusion or exclusion of travel time from the covariates does not affect the results of the primary GBM and PSM analyses.


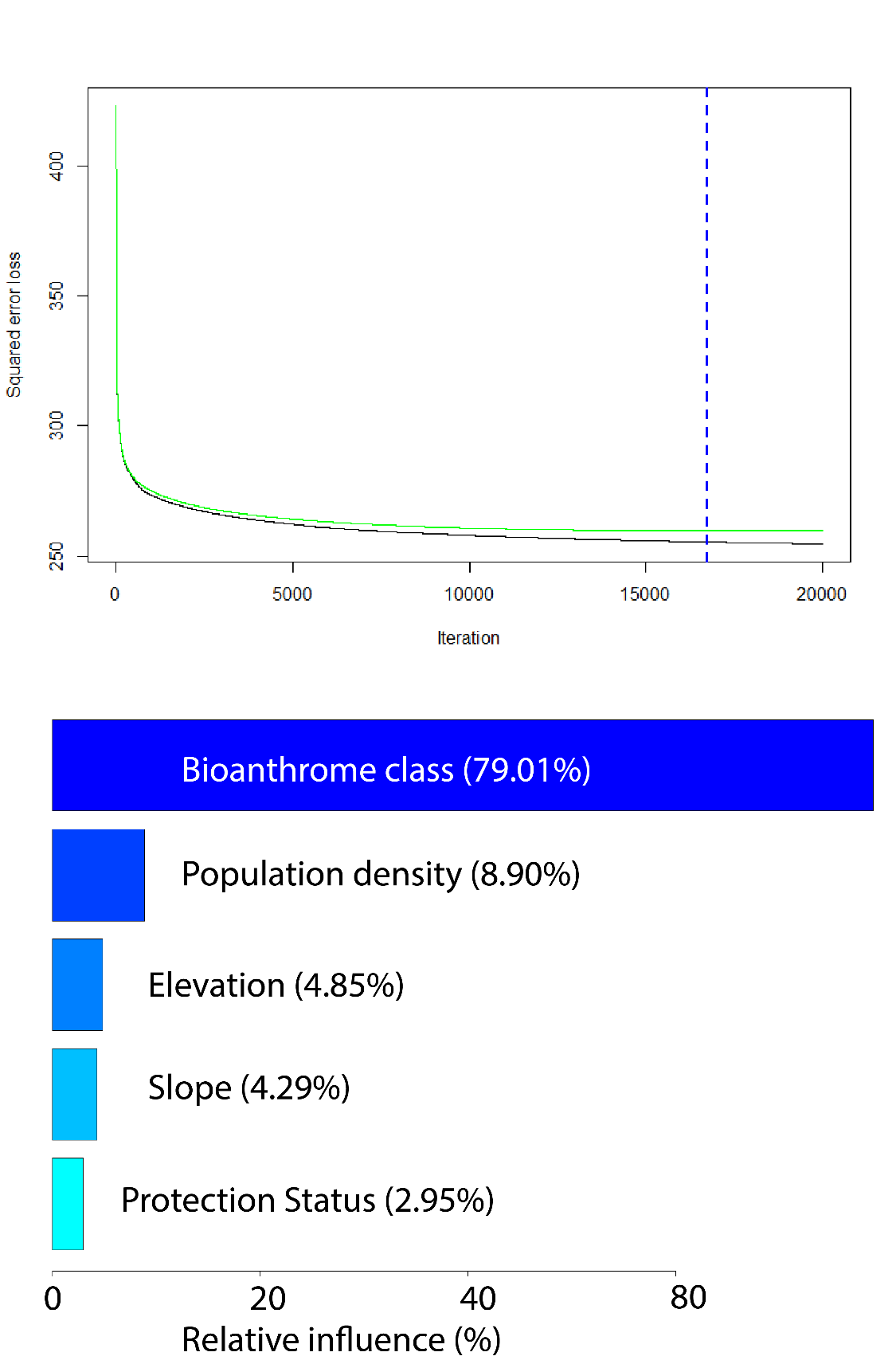


**Appendix S17.** Performance of Generalized Boosted Model with 20,000 trees with no travel time to cities as covariate explaining species loss. Compare to Appendix S9 for reference to the outcome of the primary analysis. The inclusion or exclusion of travel time from the covariates does not affect the results of the primary GBM analysis.

**
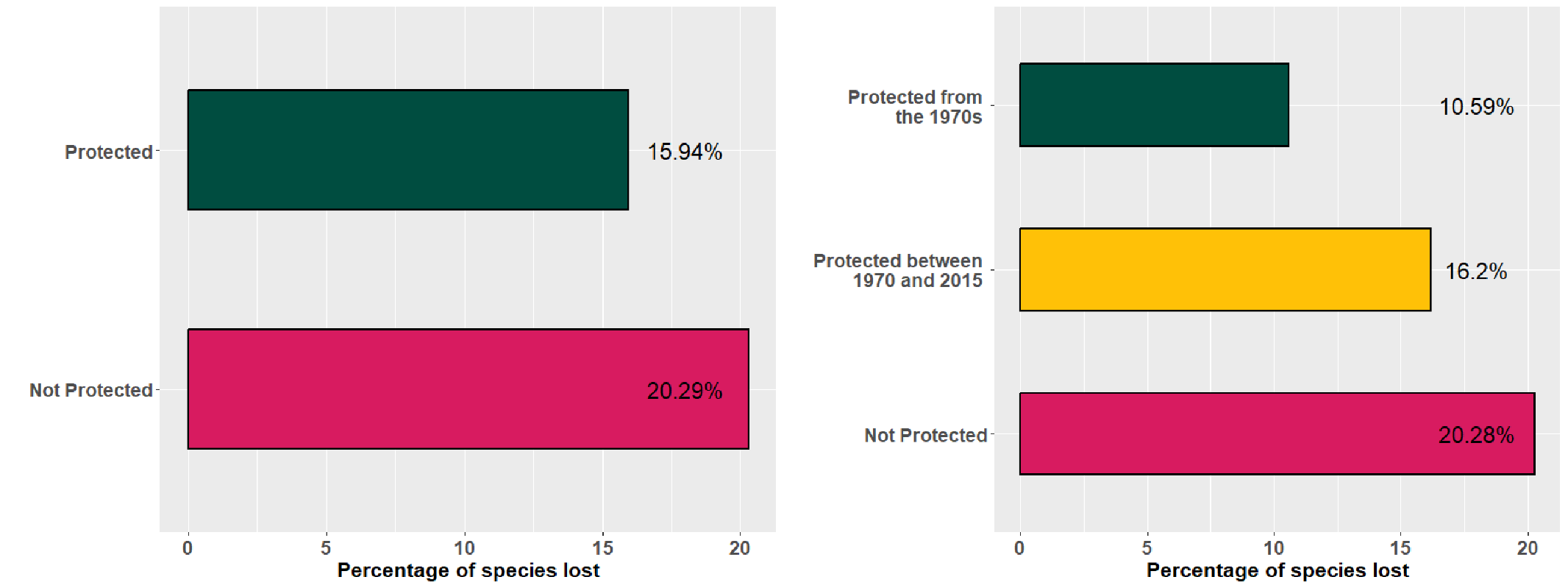
**

**Appendix S18.** Differences in rates of species loss across matched landscape units with different protection status, based on Generalized Boosted Models with potential spillover areas excluded from the control region. Differences in rates of species loss were compared between all protected and all unprotected units, and between unprotected units and units protected at a different time. Compare to Figure 2A for reference to the outcome of the primary analysis. The inclusion of exclusion of buffer zones where potential spillover may occur does not affect the results of the primary GBM analysis.


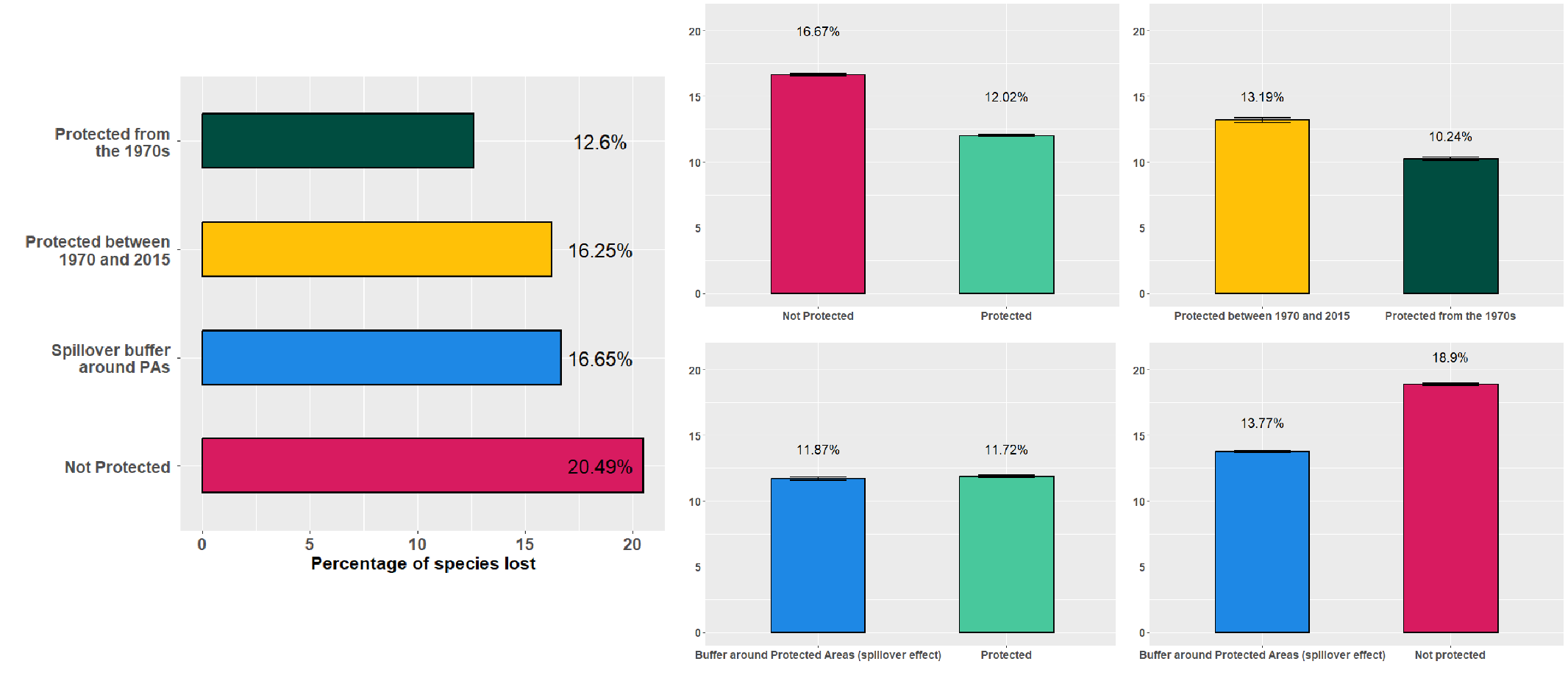
**Appendix S19.** Differences in rates of species loss across matched landscape units with different protection status, based on Generalized Boosted Models and Propensity Score Matching with no PAs with boundaries defined as a circular area around a centroid point. Compare to Figure 2 for reference to the outcome of the primary analysis. The inclusion or exclusion of travel time from the covariates does not affect the results of the primary GBM and PSM analyses.
